# Light and electron microscopy continuum-resolution imaging of 3D cell cultures

**DOI:** 10.1016/j.devcel.2023.03.001

**Published:** 2023-04-10

**Authors:** Edoardo D’Imprima, Marta Garcia Montero, Sylwia Gawrzak, Paolo Ronchi, Ievgeniia Zagoriy, Yannick Schwab, Martin Jechlinger, Julia Mahamid

**Affiliations:** 1 Structural and Computational Biology Unit, European Molecular Biology Laboratory, Meyerhofstrasse 1, 69117 Heidelberg, Germany; 2 Cell Biology and Biophysics Unit, European Molecular Biology Laboratory, Meyerhofstrasse 1, 69117 Heidelberg, Germany; 3 Electron Microscopy Core Facility, European Molecular Biology Laboratory, Meyerhofstrasse 1, 69117 Heidelberg, Germany

**Keywords:** FIB-SEM, Volume EM, CLEM, patient-derived organoids, cryo-confocal light microscopy, high-pressure freezing, Deep-learning image segmentation

## Abstract

3D cell cultures, in particular organoids, are emerging models in the investigation of healthy or diseased tissues. Understanding the complex cellular sociology in organoids requires integration of imaging modalities across spatial and temporal scales. We present a multi-scale imaging approach that traverses millimeter-scale live-cell light microscopy to nanometer-scale volume electron microscopy by performing 3D cell cultures in a single carrier that is amenable to all imaging steps. This allows for following organoids’ growth, probing their morphology with fluorescent markers, identifying areas of interest, and analyzing their 3D ultrastructure. We demonstrate this workflow on mouse and human 3D cultures and use automated image segmentation to annotate and quantitatively analyze subcellular structures in patient-derived colorectal cancer organoids. Our analyses identify local organization of diffraction-limited cell junctions in compact and polarized epithelia. The continuum-resolution imaging pipeline is thus suited to fostering basic and translational organoid research by simultaneously exploiting the advantages of light and electron microscopy.

## Highlights

• 3D cell cultures grown in sample carriers are amenable to high-pressure freezing (HPF)
• 3D cultures in HPF carriers allow for drug treatment and live-cell imaging
• Demonstrate correlative light and volume electron microscopy of 3D cell cultures
• Image segmentation links different subcellular organization and epithelium polarity

## Graphical abstract

**Figure:**
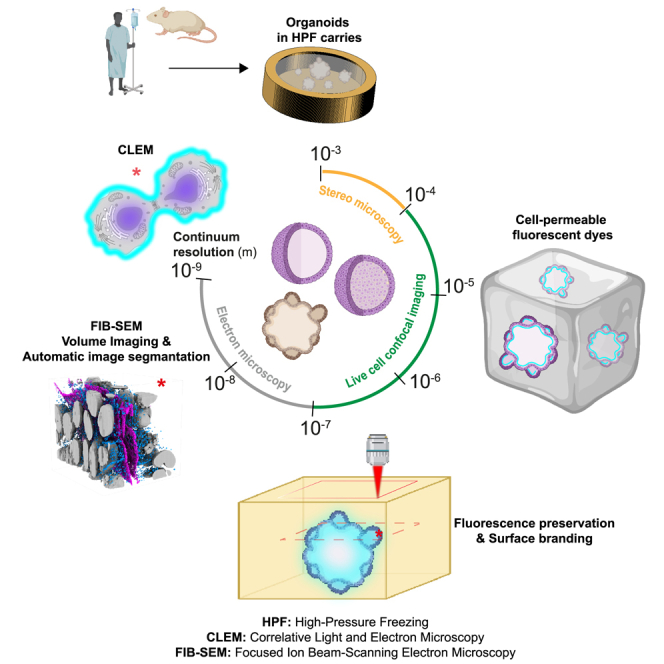

## Teaser

D’Imprima, Garcia Montero, Gawrzak, et al. develop protocols for growing mouse and human 3D cell cultures in high-pressure freezing carriers, enabling uninterrupted imaging from the light microscopy millimeter scale of entire organoids to the nanometer-scale volume electron microscopy that captures subcellular ultrastructure. The authors thereby provide a 3D multi-scale visualization of cancer disease models.

## Introduction

Cell culture systems are indispensable for a wide range of basic and pre-clinical studies. When modeling tissue-specific processes or multifaceted diseases like cancer, conventional two-dimensional (2D) cell cultures largely fail to recapitulate the complexity and cellular heterogeneity of the normal and cancerous tissue.^1^ Conversely, three-dimensional (3D) cell cultures can deliver more accurate representations of cell-cell and cell-extracellular matrix (ECM) interactions, cell communication,^2^ and cell division.^3^ 3D cancer culture models allow for the studying of tumor microenvironment, cell heterogeneity and invasion,^4^^,^^5^ as well as the effect of anti-cancer drugs.^6^ Thus, patient- or cell line-derived 3D cultures bridge the gap between simplified 2D models and organismal models that are more expensive and inaccessible to many imaging or high-throughput methods. 3D cultures can be established by anchorage-independent cell suspensions, scaffold-based approaches (natural laminin-rich hydrogels [Matrigel^7^] or synthetic ECM), tissue slices, or air-liquid interfaces.^8^^,^^9^ Commonly, 3D cultures are derived from immortalized cell lines, induced pluripotent stem cells, embryonic stem cells, or primary cells dissociated from animal or human tissues. When grown in 3D, immortalized cell lines can form hollow spheres or solid spheroids, depending on their respective epithelial polarization phenotypes. Organoids are derived from stem cells or primary cells from healthy or diseased tissues and reflect the respective tissue organization in 3D culture.^10^^,^^11^

Imaging is a powerful tool to study the complex 3D cellular sociology in organoids. Bright-field microscopy enables long-term imaging of organoid development at micrometer scale with minimal phototoxicity.^12^ Confocal microscopy and light-sheet microscopy permit the study of specific cellular processes at higher resolution using fluorescent reporters for cell types, organelles, or proteins,^13^ or using cell-permeable dyes.^14^ However, the inherent opaque nature of large organoid cultures (>200 μm) requires optical clearing.^15^ To ensure effective labeling and imaging, organoids are commonly extracted from the Matrigel.^16^ Further, immunolabeling-based fluorescence necessitates sample permeabilization. Both approaches require chemical fixation that precludes live-cell imaging.^17^ Ultrastructural detail at the nanometer scale is commonly achieved with 2D imaging of ultrathin sections by transmission electron microscopy (TEM).^18^ The biggest limitation of this technique, when imaging 3D cell cultures, is the lack of volumetric information that is essential to appreciate multicellular organization in space. Volume EM via focused ion beam-scanning electron microscopy (FIB-SEM) provides a solution to this problem. The method involves progressive specimen slicing by FIB surface ablation and iterative SEM imaging.^19^^,^^20^^,^^21^ FIB-SEM thus generates a series of images that can cover a relatively wide field of view at nanometer-scale resolution, and extensive z-slicing yields relatively large volumes of 3D ultrastructural information.^20^^,^^22^

However, none of these imaging methods alone can provide a complete functional and structural understanding of cells in the context of the complex multicellular environment of organoids. To alleviate such shortcomings, correlative approaches that combine light- and electron-based imaging modalities have been developed.^19^^,^^23^^,^^24^ Yet, their implementation in organoid research has remained limited owing to the diverse requirements these imaging modes have, and which are rarely met by one single-specimen preparation; established EM specimen preparation requires sample cross-linking by chemical fixation at room temperature, leading to disruption of the cells’ fine ultrastructure.^25^ Cryogenic fixation instead uses low temperatures to stabilize the sample under fully hydrated physiological conditions. Freezing must retain the water molecules in an amorphous state in a process called vitrification.^26^ For larger objects, including 3D cell cultures, vitrification is achievable by high-pressure freezing (HPF).^27^ Here, samples that are up to 200 μm thick are sandwiched between two metal disks, known as planchets or HPF carriers. Then, the specimen that is protected between the metal carriers is pressurized to 2,000 bar and cooled by liquid nitrogen within 200 ms. For room temperature EM, the cryo-fixed samples are subjected to freeze-substitution and stained with heavy metals to provide contrast in EM.^28^ A variation of this procedure reduces the amount of metals in the freeze-substitution cocktail to only 0.1% uranyl acetate, allowing preservation of fluorescence for targeting of specific regions of interest (ROIs) in large sample volumes by fluorescence imaging after embedding,^23^^,^^29^ while still providing sufficient contrast for FIB-SEM imaging.^30^ Such preparation methods are easily applicable for specimens that are amenable to manual handling for cryo-fixation, including small model organisms or dissected tissues. However, the fragility of 3D organoids grown in soft matrices precludes such manipulation. It is therefore beneficial to perform the 3D cell cultures and live imaging in sample carriers that are directly amenable to cryo-fixation. Here, we show that it is possible to perform 3D cultures in common HPF carriers coated with a biocompatible metal, and we develop a seamless workflow to image the cultures from their initial development to high-resolution FIB-SEM.

## Design

Compared with 2D cell cultures, organoids allow for a better model of cellular heterogeneity in healthy and diseased epithelia. Their inherent complex 3D structure requires the use of multiple imaging methods to capture disease development across scales. It is therefore beneficial to directly correlate non-invasive light microscopy that is essential for probing organoid development with high-resolution EM to image subcellular structures. We choose HPF carriers as an economical, readily available, and biocompatible carrier amenable to all the imaging modalities to minimize manual handling and perturbation of fragile 3D organoids grown in soft matrices. We opted for FIB-SEM volume imaging to capture in 3D diffraction-limited subcellular structures and probed their heterogeneity in the multicellular context of patient-derived colorectal cancer organoids. Finally, we employed trainable deep-learning automatic image segmentation to infer quantitative information from the large amount of EM data.

## Results

### Establishing a multi-scale imaging pipeline

**Figure 1:**
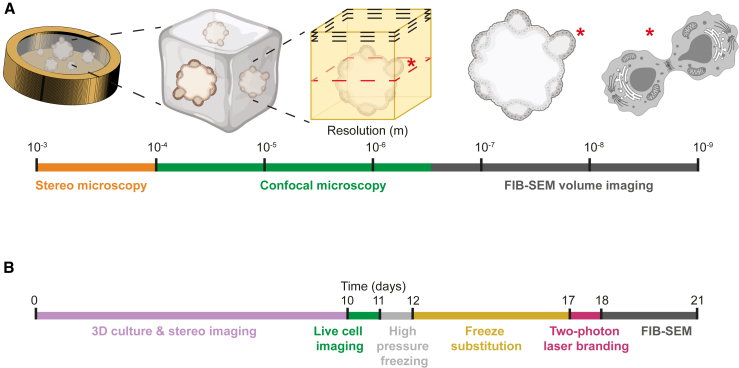
3D cell cultures in HPF carriers enable correlative imaging from the millimeter to the nanometer scale (A) A multi-scale imaging pipeline of 3D cell cultures in HPF carriers encompasses millimeter-scale stereo light microscopy, 3D confocal fluorescence microscopy prior to and following cryo-fixation, and nanometer-scale FIB-SEM volume imaging. Asterisks indicate single cells that can be targeted and followed throughout the imaging pipeline. (B) Timeline of 3D cell culture for room-temperature sample preparation and imaging.

In multimodal imaging pipelines, EM has the most stringent requirements for specimen preparation due to its high resolving power. HPF is the only approach to preserve the ultrastructure of multicellular samples. We tested whether common gold-coated copper HPF carriers as readily available supports are compatible with long-term 3D cell culture and the subsequent imaging modalities (Figure 1A). We used 200-μm-deep HPF carriers featuring a nominal 0.62 mm^3^ sample volume and 3.14 mm^2^ surface area that is exposed to the culture medium, providing a surface-to-volume ratio 2.5 times larger than our standard 3D culture conditions (90 μL Matrigel drop in tissue culture [TC] dishes).^31^ We performed the 3D cell culture directly in HPF carriers by pipetting in the recess 1–2 μL of cell suspension mixed with Matrigel (STAR Methods). The carriers were then placed in multi-well TC dishes supplied with the culture medium. To aid handling and prevent floating of the carriers, they were fixed with a droplet of Matrigel to 12 mm glass coverslips. With this setup, we could monitor the 3D cell culture growth by stereomicroscopy up to 24 days and correlate it with subsequent live-cell confocal imaging (Figures 1A and 1B). We acquired light microscopy images at single time points to monitor the organoid growth for subsequent EM. Nevertheless, acquisition of time-series confocal fluorescence microscopy (4D imaging) is technically possible using this setup. Next, we performed HPF at chosen time points of the culture. Freeze-substitution, heavy metal staining that retains fluorescence signal and Lowicryl HM20 resin embedding were carried out in preparation for room-temperature FIB-SEM.^30^ The resin block was then imaged by confocal fluorescence microscopy to relocate features previously identified in the specimen by live-cell imaging and to define ROIs. We generated landmarks on the resin block surface by two-photon laser ablation to facilitate identification of the ROIs and guide their high-resolution imaging in subsequent FIB-SEM,^30^ resulting in an imaging pipeline covering all spatial scales from 10^−3^ to 10^−9^ m on the same organoids. This pipeline requires up to 10 days, with the freeze-substitution being the longest step (Figure 1B). In summary, transferring fragile organoids into HPF carriers is a cumbersome procedure involving their removal from the ECM,^32^ which can alter the organoids’ morphology and prevents image registration pre and post cryo-fixation. Thus, by establishing 3D cultures directly in HPF carriers, sample handling and all imaging steps require minimal manual intervention.

### HPF carriers are compatible with diverse 3D cell culture models

**Figure 2:**
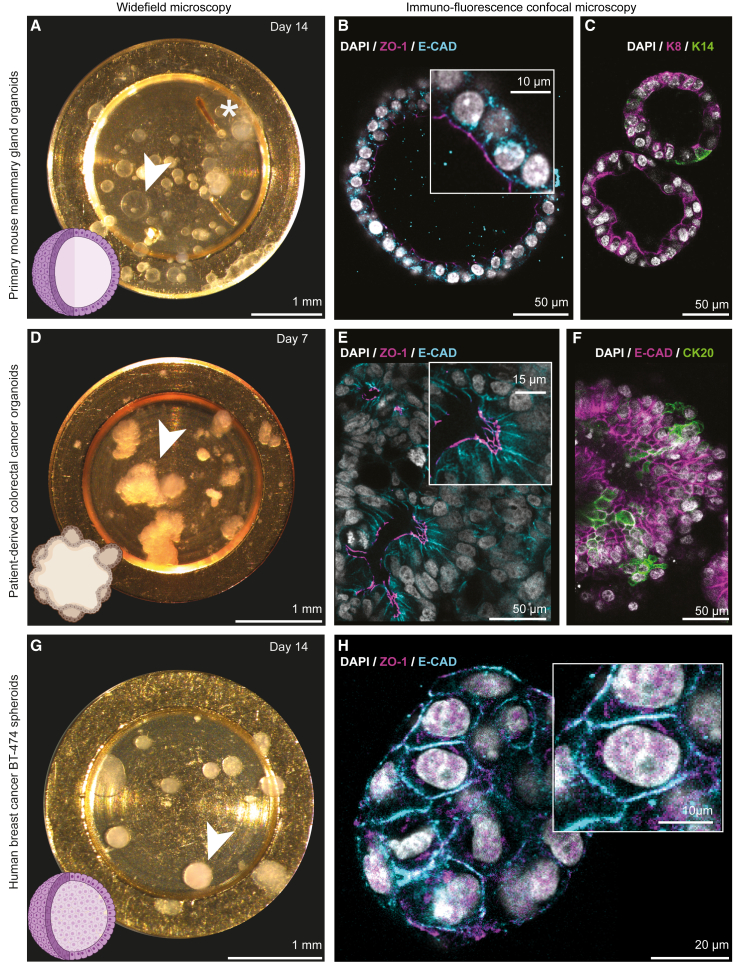
Preservation of organoid-specific 3D multicellular morphologies in HPF carriers (A–C) Mouse mammary gland organoids. (D–F) Patient-derived colorectal cancer organoids. (G and H) BT-474 human breast cancer spheroids. In (A), (D), and (F), stereomicroscopy of mouse mammary gland organoids, patient-derived colorectal cancer organoids, and BT-474 human breast cancer spheroids growth in HPF carriers at the indicated time point of the culture. Arrowheads indicate single organoids. Asterisk in (A) indicates marks that can be applied to the bottom of the HPF carriers with blunt-tip tweezers prior to Matrigel deposition to assist imaging by different light microscopes. Bottom left: schematic of organoid morphology. In (B), (E), and (H), immunostaining of polarity markers: the cell-adhesion protein epithelial cadherin (E-CAD, cyan) and tight-junction protein zonula occludens-1 (ZO-1, magenta). Cell nuclei stained with DAPI (white). Insets: details of cell-cell and cell-lumen interfaces. In (C), immunostaining with tissue-specific markers: luminal keratin 8 (K8, magenta) and basal/myoepithelial keratin 14 (K14, green). In (F), immunostaining with tissue-specific markers: epithelial cadherin (E-CAD) and cytokeratin 20 (CK20, green), a colon cell marker. See also Figure S1.

To validate the compatibility of the developed pipeline for organoid research, we examined three types of 3D cell cultures: models of primary healthy and tumorigenic (under doxycycline oncogene induction) epithelia derived from mouse mammary glands, patient-derived human colorectal cancer, and a human breast cancer cell line (Figures 2 and S1; STAR Methods). We grew our model of healthy mammary gland organoids from single-cell suspensions derived from transgenic mice expressing H2B-mCherry as a nuclear marker. Within 14 days, mammary gland organoids grown in the HPF carriers displayed the expected single layer of epithelial cells arranged around a lumen, similar to standard culture conditions (Figures 2A, 2B, S1A, and S1B).^33^ The H2B-mCherry reporter allowed us to inspect the organoids’ distribution and morphology over several days of culture (Figure S1E). Immunofluorescent staining for the apical-basal cell polarity marker epithelial cadherin (E-CAD) and the tight-junction protein zonula occludens-1 (ZO-1) showed that they localized in the apical cell membranes that line the lumen, while the basal side faces the ECM in both HPF carriers (Figure 2B) and TC dishes (Figure S1B), confirming preservation of polarized epithelia characteristics. In addition, immunostaining of tissue-specific differentiation markers on fixed samples in the HPF carriers showed that the organoids are predominantly composed of luminal mammary epithelial cells expressing keratin 8 (K8) and a few basal/myoepithelial mammary cells expressing keratin 14 (K14) (Figure 2C). Moreover, activation of *c-myc* oncogene expression by doxycycline addition in the HPF carriers leads to the development of solid tumorigenic organoids, reminiscent of the tumor-induced organoids grown on dishes^33^ (Figures S1H–S1K).

Patient-derived colorectal cancer organoids grow within a week as irregularly shaped clusters and display similar morphology in HPF carriers and TC dishes (Figures 2D and S1C). Fluorescence live-cell confocal imaging of the organoids in HPF carriers stained with the nuclear dye Hoechst-33342 showed multiple cell layers with small lumina (Figure S1F). Immunofluorescent staining in both HPF carriers (Figure 2E) and TC dishes (Figure S1D) for E-CAD and ZO-1 showed that the expected apical-basal cell polarity is recapitulated in both culture conditions. Concurrently, the organoids expressed E-CAD and the cytokeratin 20 (CK20) marker specific for colon tissue^34^ (Figure 2F).

The human breast cancer cell line BT-474 develops as regular-shaped spheroids when embedded in Matrigel and cultured in HPF carriers for up to 14 days (Figure 2G). Confocal live-cell imaging confirmed the formation of solid spheres without lumina in HPF carriers (Figure S1G), and these do not display apical-basal cell polarity (Figure 2H).^35^

Thus, the examined types of 3D cell cultures grown in HPF carriers display morphologies, composition, and cell polarity reminiscent of organoids grown on TC dishes. Therefore, HPF carriers are applicable for a range of 3D cell cultures and are compatible with confocal microscopy performed on live and fixed organoids as the first step in a multi-scale imaging pipeline.

### Small-molecule live dyes for correlative imaging of 3D cell cultures

**Figure 3:**
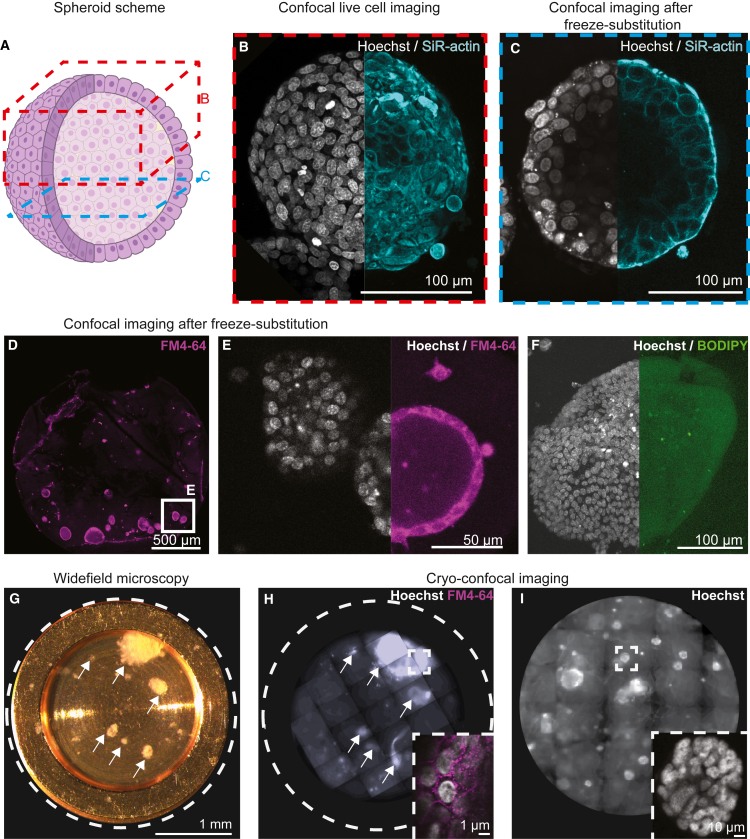
Preservation of fluorescent live dyes in cryo-fixation and freeze-substitution (A) Schematic representation of a BT-474 human breast cancer spheroid. Locations of confocal imaging before (red, B) and after (blue, C) freezing and freeze-substitution are indicated. (B) Maximum intensity projection of the live spheroid confocal volume stained with live dyes for detection of nuclei (Hoechst-33342, grayscale) and F-actin (SiR-actin, cyan). (C) A confocal plane of the exposed resin block surface. (D and E) Confocal imaging of primary mouse mammary gland organoids stained with membrane and nuclear live dyes (FM4-64 in magenta and Hoechsts-33342 in grayscale, respectively) after freeze-substitution. (F) Tumor-induced mouse organoid stained for nuclei (Hoechst-33342, grayscale) and lipid-droplet (BODIPY 497/503, green). (G) Light microscopy of patient-derived colorectal cancer organoids (white arrows) before high-pressure freezing. (H) Maximum intensity projection tiled scan cryo-light microscopy of the carrier in (G) after high-pressure freezing. Inset: organoid in (H) with nuclear (Hoechst-33342, grayscale) and membrane (FM4-64, magenta) live dyes. (I) Cryo-light microscopy of BT-474 spheroids. Inset: organoid in (I) with nuclear (Hoechst-33342, grayscale) live dye. See also Figure S2.

Genetically engineered cell lines or animal models are frequently used to prepare 3D cell cultures with fusion proteins as fluorescent reporters. The production of transgenic reporters is time-consuming and may be impractical for speedy translational molecular medicine using patient-derived organoids, although recent publications show promising results.^36^ In Figure 2, we demonstrated the applicability of HPF carriers for culturing organoids derived from transgenic mice with or without fluorescent reporters and two human-derived systems that lack genetically encoded fluorescence. For the latter, we investigated the possibility of using cell-permeable fluorescent live dyes as an alternative solution. However, small-molecule live dyes are often incompatible with fixation and dehydration procedures required for EM.^37^^,^^38^ Thus, we tested four common live dyes with different chemical properties: we used a combination of Hoechst-33342 to mark cell nuclei and one of the 3 dyes SiR-actin, FM4-64, and BODIPY 493/503. All live dyes infiltrated organoids grown in HPF carriers, allowing for live imaging of cell nuclei, of SiR-actin stained cortex in human BT-474 spheroids (Figures 3 and S2A–S2C) and in mammary gland organoids (Figures S2D–S2F), of BODIPY 493/503 stained lipid droplets in mammary gland tumor organoids (Figures S2G–S2I), and of cell membranes stained with FM4-64 in patient-derived colorectal cancer organoids (Figures S2J–S2L). In agreement with their chemical properties, hydrophilic dyes (Hoechsts-33342 and SiR-actin; Figures 3B and 3C) were preserved following freeze-substitution. The fluorescence signal of the membrane marker FM4-64 appeared diffuse after freeze-substitution but permitted discerning cell boundaries to some extent (Figures 3D and 3E). The hydrophobic lipid droplet marker BODIPY 493/503 was completely removed during the washing step with organic solvents in freeze-substitution (Figure 3F). Conversely, HPF alone did not affect the fluorescence signal of lipophilic dyes as determined by direct imaging of HPF samples with a cryo-confocal microscope (Figures 3G–3I; STAR Methods). Thus, live dyes present an alternative for genetic tagging in fluorescence-based correlative imaging in 3D cultures.

### FIB-SEM volume imaging of 3D organoids

**Figure 4:**
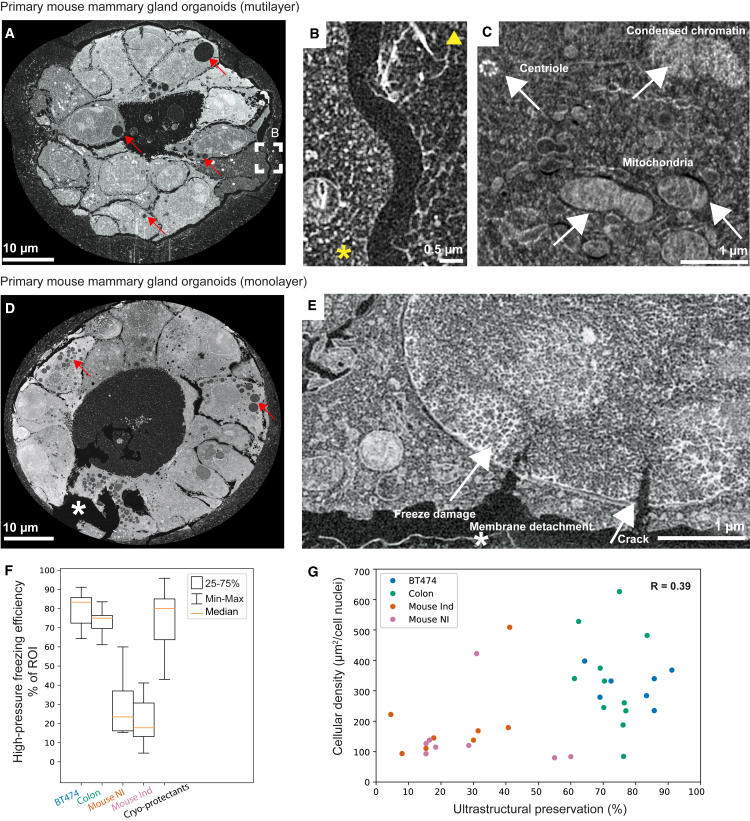
Assessment of structural preservation of 3D cell cultures (A–C) FIB-SEM of multilayer mouse organoid after 7 days of culture, showing details of the cytoplasm characteristic freezing (left, asterisk) versus Matrigel (right, arrowhead) (B) , and a well-preserved cell with condensed chromatin, centriole, and mitochondria (C). (D and E) Monolayer organoids show freezing damage and extensive cracks, with membrane detachment. Red arrows in (A) and (D) highlight cell vacuoles characteristic of mouse mammary gland organoids. (F and G) Quantification of HPF efficiency by thin section TEM and its correlation with cell density in organoid (G) for human breast spheroids (BT474, n = 7 spheroids, 1,619 examined tiles), human colorectal cancer organoids (colon, n = 11 organoids, 823 tiles), doxycycline-induced tumorigenic (mouse Ind, n = 8 organoids, 430 tiles), and healthy (mouse NI, n = 8 organoids, 596 tiles) mouse mammary gland organoids that were additionally supplemented with cryo-protectants (cryo-protectants, n = 6 organoids, 450 tiles). In (F), orange line, median; box, 25%–75% interval; whiskers, minimum and maximum. In (G), R is the Pearson correlation coefficient. Further details in the STAR Methods. See also Figures S3–S5.

Following localization of ROIs in the resin block by confocal imaging and generation of surface landmarks by laser branding in the same microscope, we proceeded to FIB-SEM imaging. It is known that vitrification efficiency by HPF varies from sample to sample.^39^ Ice crystals’ growth can induce organelles collapse, segregation, and aggregation of macromolecules. The EM data showed that organoids display different freezing behavior from their Matrigel embedding medium, which exhibits fiber-like features (Figures 4A and 4B). Mature mammary gland organoids developed in 6–8 days in culture, during which cells polarize and self-organize in monolayered acini. Before reaching this stage, cells are intertwined in several layers within crowded acini (lumen up to 15% of the organoid volume) with high cell packing density (Figure 4A). FIB-SEM data of such organoids generally showed good ultrastructural preservation with fine details of centrioles, mitochondria, and condensed chromatin (Figure 4C). Conversely, in mature monolayer organoids, the lumen comprises up to 60% of the organoid volume (Figures 2B and 4D). In this scenario, the polarized cells displayed typical freeze-substitution artifacts including cracks and membrane detachment at cell-cell and cell-ECM interfaces (Figures 4D and 4E) due to resin polymerization-induced shrinkage. Freezing damage is evident in nuclei and in the cytosol (Figure 4E), wherein dehydrated biological material segregates to form branched structures that grow from water crystal nucleation points and spread through the surrounding sample.

Despite the inherent heterogeneity of freezing quality expected in HPF, our preliminary observation of early multilayered versus late monolayered mammary gland organoids suggested that the preservation of the cellular ultrastructure improved with increased local density of cells within the organoids and reduced size of the lumen. To provide a semi-quantitative description of the freezing quality, we prepared thin sections from the HPF 3D cultures for TEM to image larger areas of the different organoids and to probe diverse and distant regions across the HPF carriers. We prepared thin sections from healthy (mouse NI) and doxycycline-induced tumorigenic (mouse Ind) mouse mammary gland organoids, human spheroids (BT-474), and patient-derived colorectal cancer organoids. The sections were cut orthogonal to the HPF carrier surface, aiming for organoids located in the bulk of the Matrigel (Figure S3A). We imaged six to ten organoids per sample, spaced between 90 and 400 μm from the edge of the HPF carrier toward its center, and surveyed large areas by tiling multiple TEM imaged spots (Figures S3A and S3B). We visually inspected each tile for the presence of freeze damage artifacts and scored the quality of structural preservation. Overall, our survey shows that compact organoids like human colorectal cancer achieve high ultrastructural preservation, with a minimum of 60% of structurally intact regions and median of 83%, or human breast cancer spheroids with a minimum of 60% and median of 75% (Figure 4F). Conversely, we obtained less consistent success for mouse organoids with large lumina (17%–23% median ultrastructural preservation, Figure 4F). More specifically, human colorectal cancer organoids grow as large and compact structures that protrude into the Matrigel with multiple branches featuring one lumen (Figure S3C). Unlike the mouse organoids, human colorectal organoids showed better preserved cell ultrastructure and a larger number of cells with little or no freezing damage (Figures 4F and S3B). Human breast cancer spheroid represented the densest cell system that is devoid of a lumen and exhibited minimal freezing damage, which was restricted to the center of the sample, as expected from theoretical considerations of heat transfer during HPF (Figures 4F and S4). Structural damage was confined to cell nuclei, and the occasional cytosolic segregation was restricted to one or two cell layers at the spheroid center (Figure S4). Finally, we estimated the cell packing density for all systems as the ratio between the organoids’ total area divided by the number of cells, approximated by counting the nuclei in the TEM sections. Our data suggest that more compact organoids suffer less freeze damage despite the use of an identical embedding matrix and carrier dimensions (Figure 4G).

Optimization of HPF conditions is generally required for each specimen type.^40^ This typically consists of embedding or infiltrating the sample with release agents like 1-hexadecene or lecithin and with cryo-protectants such as glycerol, dimethyl sulfoxide (DMSO), bovine serum albumin (BSA), yeast paste, gelatin, polyvinylpyrrolidone, dextran, sucrose, and Ficoll. We therefore sought to improve the vitrification of the least preserved 3D culture sample encountered, namely mammary gland mouse organoids, by introducing cryo-protectants prior to cryo-fixation. We found that dipping the HPF carriers for 1 min before HPF in Cellbanker 2, a common medium for preserving cells in liquid nitrogen (STAR Methods), or in 20% Ficoll (70,000 MW) dissolved in cell culture medium improved the sample quality after HPF (Figure S5). Quantification of the ultrastructural preservation showed improvement from ∼25% to ∼77% (median values, Figure 4F).

In summary, Matrigel-embedded organoids are challenging samples for successful HPF because of the high-water content within the embedding medium. Nevertheless, Matrigel provides the necessary physiological environment for 3D cell cultures. We found that organoids with local high-density cell packing are more resilient against freeze damage and provide adequate ultrastructural preservation. The different organoid systems require optimization in preparation for HPF, which can be achieved by careful selection of additives.

### Multimodal imaging of 3D organoids with continuum spatial resolution

**Figure 5:**
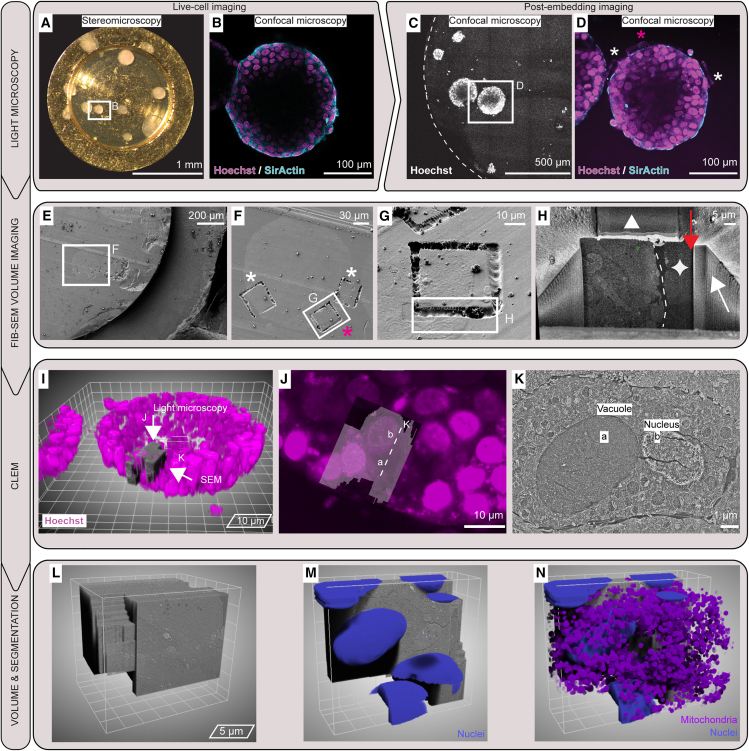
Correlative multi-scale imaging of 3D cell cultures (A) Stereomicroscopy of BT-474 human breast cancer spheroid cultured in HPF carrier. (B) Live-cell confocal slice of the indicated spheroid in (A), showing nuclei (Hoechst-33342, magenta) and F-actin (SiR-actin, cyan). (C) Maximum intensity projection tiled scan of the specimen from (A) after HPF and freeze-substitution. Frame indicates the spheroid imaged in (B) and enlarged in (D). (D) Confocal plane after freeze-substitution. Surface laser brandings were introduced at positions of interest (white asterisks) to guide FIB-SEM volume imaging (magenta asterisk). (E) SEM view of the sample in (D). (F–H) Targeted FIB-SEM acquisition. (F) Brandings (white asterisks) and FIB-SEM target (magenta asterisk) before (G) and after trench milling (H), showing the organoid outer edge (white dash) next to the embedding Matrigel (white star). Details of the acquired volume are shown in Video S1. White triangle indicates the protective platinum layer. Red and white arrows indicate the milling direction and the progression, respectively. (I–K) Correlated light and electron volume imaging of targeted cells from the organoid in (H) (magenta asterisk in D and F). (I) CLEM overlay of the nuclear fluorescence (Hoechst, magenta) of the organoid in (B)–(D) (mesh size 10 μm) and the targeted FIB-SEM acquisition in (H) (gray). Top arrow indicates the fluorescence imaging direction shown in (J). Side arrow indicates the imaging direction in the FIB-SEM acquisition shown in (K). Post-embedding fluorescence and SEM imaging planes are displayed in (J) and (K), respectively, where the correlated location of a vacuole (a) and nucleus (b) are highlighted. (L–N) 3D representation (mesh size 5 μm) of the FIB-SEM acquisition (L), and automatic segmentation of nuclei (M), combined with mitochondria (N). See also Video S1.

Using the HPF carriers as a container for 3D cell culture allows for minimal manual intervention during their development and facilitates correlation of diverse modalities to achieve continuum-resolution imaging from millimeter to nanometer scale. We demonstrate the full imaging pipeline by performing 3D cell culture of human breast cancer spheroids directly in the HPF carriers and monitoring their growth by stereomicroscopy (Figure 5A). At a chosen development stage of the culture, each carrier was stained with live dyes and transferred into 35-mm glass-bottom dishes for the acquisition of live-cell confocal volumes (Figure 5B). This provided information on the overall architecture of the 3D cell culture and allowed for selection of cells within specific organoids for higher-resolution imaging. Subsequently, the samples were high-pressure frozen and processed with a gentle freeze-substitution protocol to preserve the fluorescence signal (Figure 5C). Two-photon laser surface branding of the resin-embedded samples at selected positions identified by fluorescence aided the targeting of FIB-SEM data with micrometer precision (Figure 5D). We identified the surface branded targets in the SEM, performed metal deposition and trench preparation following established protocols (Figures 5E–5H), and acquired FIB-SEM data with isotropic sampling of 10 nm^3^/voxel (Video S1). Typically, precise correlation of light and EM (CLEM) data in 3D still requires tedious manual positioning and registration. Alternatively, dedicated hardware that integrates a wide-field light microscope into a dual-beam FIB-SEM provides means for direct registration of the data.^41^ Here, because all imaging steps were performed on the same specimen with minimal perturbation, we could precisely overlay the FIB-SEM volume data of just a few cells within the 7 × 10^6^ μm^3^ volume of fluorescence data (Figures 5I–5K). Finally, we show that the FIB-SEM data acquired from six cells within the BT-474 organoids were of sufficient contrast and quality by segmenting the nuclei and mitochondria in cells targeted for acquisition (Figures 5L–5N).

Video S1. FIB-SEM volume imaging of cells within a human breast cancer spheroid, related to Figure 5 Volume imaging of cells within the spheroid shown in Figure 5A. Labels highlight mitochondria, vacuoles and nuclei. For display purposes, the dataset was downscaled in x,y,z by half compared to the original FIB-SEM data acquired with voxels of 10x10x10 nm and the contrast adjusted by normalizing the local contrast in Fiji.

### Characterizing ultrastructural heterogeneity of tumorigenic organoids

Intratumor heterogeneity is a prominent feature of cancer and a major impediment to personalized medicine.^42^ Thus, we focused the imaging and analysis on our model of patient-derived colorectal cancer organoids that exhibited a high level of structural preservation by HPF (Figure S3). Fluorescence microscopy showed that mature organoids display regions with different cell packing, depending on the presence of branched lumina (Figures 2D, 2E, and S2L). We took advantage of this heterogeneous morphology to investigate whether and how it translates to structures at the subcellular level. We used nuclear staining (Hoechst-33342) to define three positions that differed in their tissue architectures for FIB-SEM acquisitions (Figures S6A and S6B). From those, we collected datasets of 121,000, 113,000 and 204,000 μm^3^ volumes at a sampling rate of 15 × 15 × 20 (x, y, z) nm^3^/voxel over 72 h per dataset (Figures S6A–S6D). The organoids displayed complex networks of cells: heterogeneous cell packing spaced by multiple lumina (Video S2) alternated with polarized parts of the organoids (Figure S6E; Video S3) or with highly packed epithelium morphology (Video S4). 3D volume imaging allowed us to capture cancer-related events, such as entotic cells, that are rarely observed either because of insufficient resolution or absence of specific fluorescent markers (Figure S6F).^43^ In this non-apoptotic process, some cells internalize adjacent ones and degrade them by lysosomal enzymes.^44^ This process can occur when cells struggle to scavenge nutrients from their microenvironment, particularly where tumor vasculature is either deficient or absent. Interestingly, entosis has yet to be described in colorectal cancer,^43^ and unlike previously reported,^44^ exposure to Matrigel did not inhibit the internalization process.

Video S2. FIB-SEM volume imaging of a patient-derived colorectal cancer organoid with mixed arrangement of cell packing, related to Figure 7 Related to Figures 7A, 7D (green), and acquisition position (3) in S6A. Labels highlight nuclei, microvilli, lumina, one entotic cell and cell junctions. For display purposes the dataset was downscaled in x,y,z by half compared to the original FIB-SEM data acquired with voxels of 15x15x20 nm.

**Figure 6:**
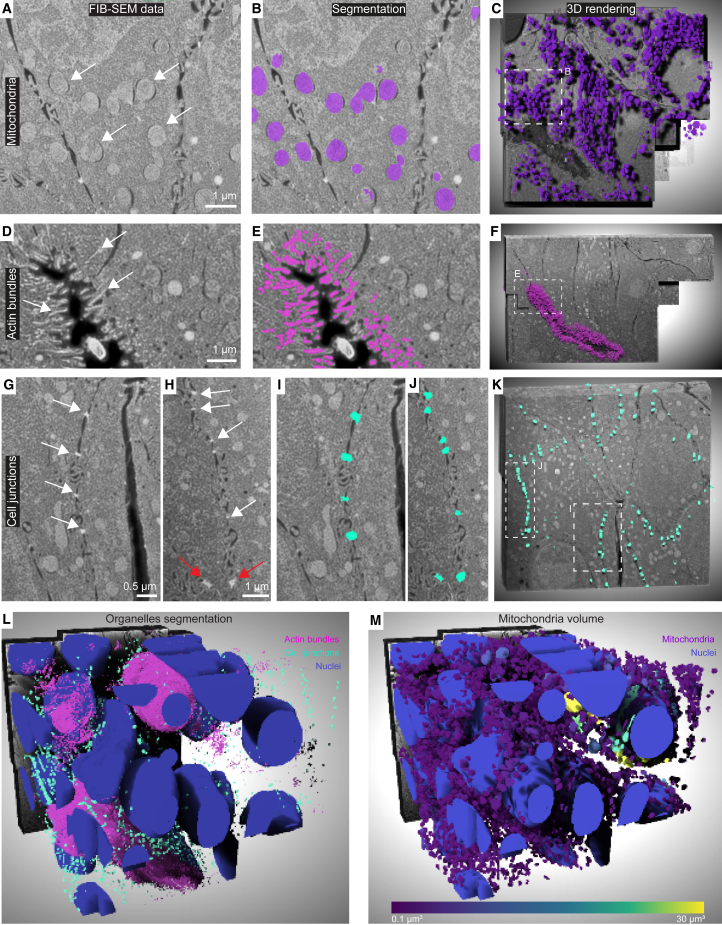
FIB-SEM volumes of patient-derived colorectal cancer organoids visualize fine ultrastructural details (A–K) Visualization and segmentation of subcellular structures. In (A), (D), (G), and (H), FIB-SEM frames showing mitochondria (A–C), actin bundles in microvilli (D–F), and cell junctions (G and H, white arrows) and desmosomes (H, red arrows) overlaid with the corresponding automatic segmentation (B, E, I, and J, in color). In (C), (F), and (K), 3D rendering of 50 consecutive frames with embossed segmentations. Frame indicates the areas shown in left panels. (L) 3D rendering of segmentations of nuclei (blue), actin bundles (magenta), and cell junctions (cyan). (M) 3D rendering of segmentation with nuclei (blue) and mitochondria colored according to their volumes (purple to yellow gradient); note the two-order of magnitude larger mitochondria around the nuclei of two cells (green-yellow). See also Figure S6 and Videos S2 and S5.

**Figure 7:**
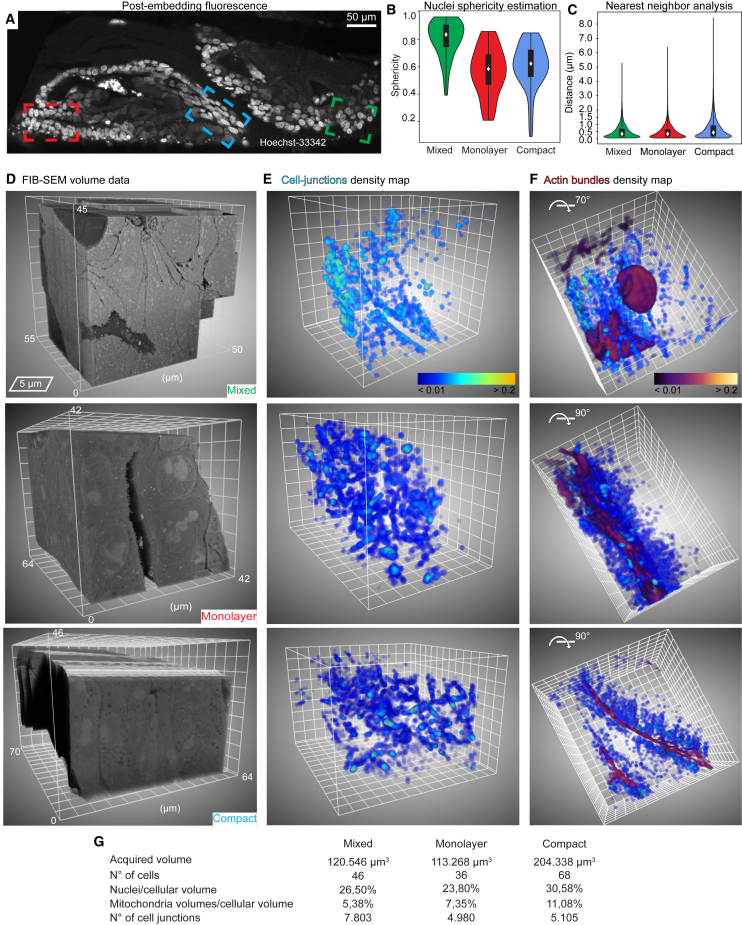
Quantitative analysis of fine cellular morphology in patient-derived colorectal cancer organoid (A) A confocal plane from patient-derived colorectal cancer organoids (volume rendered in Figure S6C). Post-embedding fluorescence shows cell nuclei stained with Hoechst. Highlighted are the FIB-SEM acquisitions corresponding to different cell packing morphologies: mixed (green), monolayer (red), and compact (blue), corresponding to positions 3, 1, and 2, respectively, in Figure S6A. (B and C) Nuclear sphericity (B) and nearest-neighbor analysis of cell junctions (C), based on the automatic segmentations exemplified in Figure 6. Box within the violins, 25%–75% data interval; diamonds, median. (D) 3D representation of the three morphologically different areas. Here, x, y, and z dimensions are shown. The first 20 μm of the mixed morphology are not displayed to aid the visualization of multiple lumina. (E) Local density maps of cell junctions based on the segmentation colored as a blue-orange gradient (mesh size 5 μm). (F) Same as in (E), rotated by 90°, and includes the actin bundles’ density maps (fire gradient). The density maps definition and calculation procedure are detailed in the STAR Methods. (G) Summary of the salient statistics of the acquired FIB-SEM volumes. See also Figure S6 and Videos S2, S3, S4, S5, S6, and S7.

Video S3. FIB-SEM volume imaging of a patient-derived colorectal cancer organoid with monolayer epithelium cell packing, related to Figures 6 and 7 Related to Figures 7A, 7D (red), and acquisition position (1) in S6A. Labels highlight lumen, nuclei, mitochondria, microvilli, condensed chromatin and cell junctions. For display purposes the dataset was downscaled in x,y,z by half compared to the original FIB-SEM data acquired with voxels of 15x15x20 nm.

Video S4. FIB-SEM volume imaging of a patient-derived colorectal cancer organoid with compact cell packing, related to Figure 7 Related to Figures 7A, 7D (blue), and acquisition position (2) in S6A. Labels highlight nuclei, mitochondria, cell junctions, lumina and microvilli. For display purposes the dataset was downscaled in x,y,z by half compared to the original FIB-SEM data acquired with voxels of 15x15x20 nm.

To provide comprehensive characterization of the heterogeneity at the subcellular level across the different organoid morphologies, we segmented cells and organelles in the FIB-SEM data. While manual annotation becomes unfeasible given the large amount of data, deep-learning algorithms offer a workable solution for automating this task. We used convolutional neural networks (CNNs) and trained instance-type segmentation networks for single cellular structures (STAR Methods). We focused on two commonly segmented targets (cell nuclei and mitochondria) that are large, have roughly defined shapes, and are highly represented in the cell volumes and two challenging fine structures (actin bundles in microvilli and cell junctions). Manual annotation was first performed on multiple subframes to train a CNN that can automatically segment a specific organelle (e.g., mitochondria) within the whole volume (Figures 6A–6C). Of special relevance for cancer development are the interactions between the cortical cytoskeleton and cell junctions.^45^ Junctional complexes play a role in the oncogenic signaling cascade that regulates cytoskeleton remodeling. This leads to a loss of epithelial cell polarity and tissue invasion by cancer cells in the process of epithelial-to-mesenchymal transition.^46^ Despite the low amount of stain (0.1% uranyl acetate) in our preparations, patient-derived colorectal cancer organoids featured high contrast for actin bundles and different types of cell junctions. Actin bundles were mostly evident within microvilli at the apical side of cells facing lumina, appearing as ∼1 μm-long fibers or high-contrast dots when cut parallel or perpendicular to the image plane, respectively (Figure 6D; Videos S2, S3, and S4). The trained CNN segmented scattered actin bundles within cells and those that form the basis of luminal microvilli (Figures 6E and 6F; Videos S5, S6, and S7). Cell junctions are inherently smaller and less represented in the data. Although the FIB-SEM resolution was sufficient to distinguish between tight junction (Figures 6G and 6H, white arrows) and larger desmosomes (Figure 6H, red arrows), the CNN could not segment them separately due to their insufficient pixel sampling. Nonetheless, a dedicated CNN segmented the detectable cell junctions in all the EM data (Figures 6I, 6J and 6K). A 3D rendering of the cell nuclei, actin bundles, and cell junctions of one of the acquired volumes (area 3 in Figure S6A) shows the complexity of the organelle arrangement in colorectal cancer organoids (Figure 6L).

Video S5. FIB-SEM volume segmentation of a patient-derived colorectal cancer organoid with mixed arrangement of cell packing, related to Figure 7 2D rendering of the volume in Figure 7 (green) and Video S2 showing the FIB-SEM data overlayed with labels of cell nuclei (light blue), entotic cell (gold), mitochondria (pink), actin bundles (magenta) and cell junctions (cyan). The segmentation is then rendered in 3D without FIB-SEM data overlayed.

Video S6. FIB-SEM volume segmentation of a patient-derived colorectal cancer organoid with monolayer epithelium cell arrangement, related to Figures 6 and 7 2D rendering of the volume in Figures 7 (red) and S6E and Video S3 showing the FIB-SEM data overlayed with labels of cell nuclei (light blue), mitochondria (pink), actin bundles in microvilli (magenta) and cell junctions (cyan). The segmentation is then rendered in 3D without FIB-SEM data overlayed.

Video S7. FIB-SEM volume segmentation of a patient-derived colorectal cancer organoid with compact cell packing morphology, related to Figure 7 2D rendering of the volume in Figure 7 (blue) and Video S4 showing the FIB-SEM data, overlayed with labels of cell nuclei (light blue), mitochondria (pink), actin bundles in microvilli (magenta) and cell junctions (cyan). The segmentation is then rendered in 3D without FIB-SEM data overlayed.

The segmentations of the different structures permit statistical analysis of the organelles for quantitative characterization of the organoids. For example, mitochondria generally range from onion-like to highly reticular shapes, whereas their dimensions are roughly uniform in non-diseased cells.^47^^,^^48^^,^^49^ When scoring mitochondria according to their volume, we found that they overall showed consistent volumes, with the exception of two cells with mitochondria that were two orders of magnitude larger than the others (Figures 6M and area 3 in S6A; Videos S2 and S5). However, we could not identify structural features in the cells or their environment that otherwise point to their different state.

We next turned to quantitatively evaluate differences in subcellular structures associated with different cell packing arrangements. Based on the fluorescence data, we refer to the different cell packing as mixed, monolayer, or compact (Figure 7). The mixed area shows heterogeneous arrangements (Figures 7A green and area 3 in S6A), a recess displays a long lumen surrounded by cell monolayers (Figures 7A red and area 2 in S6A), and the compact zone shows dense cell packing (Figures 7A blue and area 1 in S6A). It is known that healthy polarized cells in epithelia have roughly spherical nuclei and asymmetric organelle distribution in 2D cultures.^50^ It is however expected that cancer cells will exhibit more heterogeneous morphologies that have yet to be described in detail in 3D cell cultures. Indeed, the colorectal cancer cells show spherical nuclei in mixed cell packing parts of the organoids (Figure 7B), whereas monolayer or compact cell packing show spheroid-shaped nuclei (Figure 7B). This is in line with literature on 2D cell cultures where cancer cells adapt nuclear shape to account for external forces or in response to constrained space during invasion.^51^ Interestingly, the ratios between nuclei or mitochondria and cell volumes were similar in all three cell packing morphologies (Figure 7G). However, the majority of cells show nuclei with long clefts and grooves, and occasionally polynucleated cells with mitochondria intertwined between the nuclei (Videos S5, S6, and S7), demonstrating the heterogeneous nature of the organoids that can only be pertained in 3D conditions.

We then investigated potential relationships between multicellular architectures and the spatial distribution of cell junctions (Figures 7C–7F). We counted 7,803, 4,980, and 5,105 individual cell junctions in the areas with mixed, monolayer, and dense cell packing, respectively (Figure 7G). A visual inspection of the segmentations showed that the cell junctions follow the cell boundaries (Figure 6K; Videos S5, S6, and S7). Nearest-neighbor analysis of the segmented cell junctions interestingly showed that inter-junction shortest distances in all three volumes have a similar pattern, with 90% of the data falling within a 1-μm distance (Figure 7C). However, global nearest-neighbor analysis does not account for local variations. We therefore calculated density maps of the local concentration of cell junctions (Figure 7E; STAR Methods) and for the actin bundles (Figure 7F). Here, density maps of the segmented subcellular structures provide a measure of how many voxels are occupied by the structure within a specified volume. This allowed us to visually evaluate the extent of microvillar actin bundles and cell-junction co-occurrence in specific locations. The density maps indicate that local higher density of cell junctions coincides with local higher density of actin bundles that line up the organoid lumina. Our data demonstrate that more compact and polarized epithelia seal off lumina with a higher local concentration of cell junctions and microvilli, reminiscent of patterns observed in the Madin-Darby canine kidney (MDCK) cell line model using light microscopy and thin section TEM.^52^^,^^53^^,^^54^ While the molecular composition of junctional complexes and their dynamicity are well characterized in homogeneous cell line models that can establish epithelial polarity, not much is known about their 3D spatial arrangement at ultrastructural resolution in heterogeneous organoids. We thus provide a quantitative description of the local distribution of diffraction-limited cell junctions in cancer tissue models using patient-derived organoid.

## Discussion

We describe a multi-scale imaging pipeline across optical and electron microscopes that enables seamless correlative investigations in the emerging model systems of 3D cell culture. Performing the 3D culture directly in standard HPF carriers retained the expected morphology for a diverse set of model organoids (Figure 2) and guaranteed that the sample remained unperturbed from the beginning of cell seeding until HPF. This avoids the need for dedicated carriers^55^ or additional mounting media for confocal and light-sheet imaging.^56^ Thus, the use of HPF carriers enabled us to correlate the growth of the 3D cell cultures (stereomicroscopy), the visualization of multicellular architectures and cellular structures of interest (live-cell fluorescence confocal imaging) for subsequent post-embedding imaging and laser branding, to ultimately guide FIB-SEM volume imaging for ultrastructural investigations. HPF carriers further provide a high surface area to volume ratio that allows infiltration with diverse small molecules (100–1,000 Da molecular weight), including drugs like doxycycline (Figure S1). Thus, 3D cell cultures performed in HPF carriers are amenable to drug screening and treatment, with the advantage of being directly accessible to imaging by both light and EM. Because of the unspecific and broad-spectrum binding of the uranyl-acetate stain, FIB-SEM can image multiple subcellular components at nanometer-scale resolution within hundreds of thousands of cubic micrometers of a multicellular sample guided by fluorescent signal. Future efforts for integrating such approaches into high-throughput platforms for organoid research will facilitate expanding organoid phenotyping to the ultrastructural level and aid in deriving mechanistic understanding of tissue-specific processes or drug effects.

The ability to image 3D cell cultures across scales allowed us to investigate diffraction-limited structures in their multicellular context that are otherwise difficult to capture by either 2D TEM or light microscopy alone. Fluorescence signal guided the FIB-SEM acquisition of more than 10^5^ μm^3^ in a colorectal cancer organoid at ultrastructural resolution, corresponding to roughly 30–70 cells in a single imaging session. Further, automatic image segmentation provided quantitative information on the imaged data. The combination of these methods provided an ultrastructural 3D map of cell junctions. The data unveiled that cancer cells sealing off lumina in areas with different packing topologies exhibited some polarity features of healthy cells (like apical microvilli). However, 3D mapping of individual junctional complexes (desmosomes, tight and adherence junctions) indicated abnormal polarity as they seem not to always localize with luminal microvilli (Figures 7D–7F mixed cell packing; Video S5). These possibly point to cells with higher invasive and metastatic potential within different parts of the tumor. Such level of tumor heterogeneity can only be probed at ultrastructural resolution, across multiple cells within an epithelium minimally perturbed by sample preparation, thanks to the unspecific stain for EM that allows 3D imaging of all subcellular structures at once.

Beyond the scope of this study, potential applications of our pipelines are several and multidisciplinary. We envision that patient-derived tumor organoids will be amenable to ultrastructural analysis to study multicellular interaction during drug treatment, enabling a deeper understanding of drug resistance, e.g., via loss of polarity/epithelial-mesenchymal transition^57^^,^^58^^,^^59^ and therapies modulating cell-cell contacts.^60^ Co-cultures of patient-derived colorectal carcinoma organoids have been used to interrogate the effects of anaerobic microbiota on tumor progression.^61^ The use of fluorescently marked patient-derived organoids together with differentially marked associated microbial taxa allows now for the ultrastructural investigation of tissue/bacteria interactions,^62^ as well as the loss of epithelial barrier integrity.^63^

As proof of principle, we also demonstrated that it is possible to perform cryo-fluorescence light microscopy on organoids in HPF carriers (Figures 3H and 3I) toward applications in full cryogenic regime aiming to expand the resolution range to the molecular scale. However, cryo-CLEM for HPF samples still suffers from technical shortcomings, including light scattering when imaging beyond the first few micrometers from the HPF carrier surface, thus requiring development of new objectives with working distance ≥1 mm and NA >0.5. In addition, unlike room-temperature approaches, it is not possible to brand the sample surface for precise correlation with FIB-SEM imaging, although new carriers with finder patterns may provide a possible solution.^64^

In summary, the use of 3D culture technology to model tissue complexity and cellular crosstalk is becoming increasingly evident but lacks appropriate multimodal tools for comprehensive studies across spatial scales. Our multi-scale imaging pipeline achieves a resolution continuum from millimeter scale to nanometer scale and is applicable to common 3D cell cultures used in organoid and cancer research. While sample-specific optimization will be required to achieve optimal ultrastructural preservation for different organoid types, the developed pipeline and protocols could be easily adopted by EM labs that routinely practice HPF and FIB-SEM volume imaging, broadening their capability to the study of 3D cultures.

### Limitations of the study

The use of cancer organoids as model systems better recapitulates disease complexity, compared with 2D cell cultures. However, this requires longer culturing time and higher costs. Importantly, the embedding matrix medium limits sample handling and preparation for correlative studies across scales and imaging modalities. New culturing methods for higher throughput^65^ in a Matrigel-free environment^66^ will further streamline the integration of multiple imaging methods. 3D cell cultures further entail poorer ultrastructural preservation following freezing. We provide a semi-quantitative evaluation of the resilience to HPF of different organoid systems, showing that organoids with larger lumina are more prone to freeze damage and highlighting some cryo-protectants that reduce this effect.

We established that several live dyes are resilient to freeze-substitution and can therefore assist in identifying specific cells or phenotypes^13^ to study systems not easily accessible to genetic engineering. However, it is important to highlight the limits of light microscopy for large and opaque objects like organoids. Photon scattering within biological material increases with the light path through the sample, leading to dimmer fluorescence the deeper one images.^67^^,^^68^ This problem might be enhanced by limited diffusion of dyes within a compact, tight-junction sealed epithelium. Loss of fluorescence signal is particularly severe for structures with dense cell packing like spheroids and parts of colon organoids,^69^^,^^70^ further exemplified by our observation that the fluorescence signal exhibits a gradient from the core of such organoids (Figures 3C and 7A).

The durability of the FIB source and lengthy ablation time limit successful FIB-SEM imaging to a 50–60-μm depth from the specimen surface. However, one could access organoids within any depth of the HPF carrier by removing excess resin by ultramicrotomy, guided by the preserved fluorescence after freeze-substitution.^30^ In addition, a compromise exists between imaging resolution and the extent of acquired volume to achieve reasonable imaging time and throughput. While FIB-SEM imaging allows for 4–8 nm isotropic acquisitions of an entire cell, we found that when addressing a multicellular sample, 15–20 nm anisotropic sampling is a suitable compromise. However, developments in hardware hold promise to achieve ultrastructural resolution on large samples at higher througput.^71^^,^^72^

Image processing to infer quantitative information from FIB-SEM data also represents a technical limitation. Few commercial software provide teaching material, data handling interfaces, and user-friendly deep-learning capabilities, but are generally not accessible to high-performance computing on clusters for the processing of large amounts of data within a manageable time. Conversely, although current open-source software are often not easily deployed by non-experts, they offer a more scalable solution. Most importantly, open-source software foster public sharing of raw data^73^ and CNN models^74^ to prompt further development.

## STAR★Methods

### Key resources table

**Table:** 

REAGENT or RESOURCE	SOURCE	IDENTIFIER
**Antibodies**		

Rabbit anti c-MYC	Cell Signaling Technologies	Cat# 5605; RRID: AB_1903938
Rabbit anti ZO-1	Thermo Fisher	Cat# 61-7300; RRID: AB_138452
Mouse anti E-cadherin	Thermo Fisher	Cat# 13-1700; RRID: AB_86564
Rat anti Alpha6-integrin	Millipore	Cat# MAB1378; RRID: AB_2128317
Mouse anti Cytokeratin 8	DSHB	Cat# Troma-I; RRID: AB_531826
Mouse anti Cytokeratin 14	Invitrogen	Cat# MA5-11599; RRID: AB_10982092
Rabbit anti Cytokeratin 20	Abcam	Cat# ab76126; RRID: AB_1310117
Alexa 488 anti rabbit	Invitrogen	Cat# A-11034; RRID: AB_2576217
Alexa 568 anti mouse	Invitrogen	Cat# A-11031; RRID: AB_144696
Alexa 647 anti rat	Invitrogen	Cat# A-21247; RRID: AB_141778

**Chemicals, peptides, and recombinant proteins**		

Uranyl Acetate	Agar scientific	Cat# R1260A
Acetone, glass distilled	Electron Microscopy Sciences	Cat# 10016
Lowicryl HM20 kit	Polysciences, Inc.	Cat# 15924-1
Super Glue Liquid Precision Max	Loctite	N/A
Silver paint (EM-Tec Ag44 conductive silver paint)	Micro to Nano	Cat# 15-002144
Cornig® Matrigel® basement membrane matrix	Sigma	Cat# CLS354234
DMEM/F12	Lonza	Cat# BE12-719F
HEPES	ThermoFisher	Cat# J67485.AE
Penicillin Streptomycin solution	ThermoFisher	Cat# 15140122
Collagenase Type III	Worthington Biochemical Corporation	Cat# LS004180
Liberase™ TM Research Grade	Roche	Cat# 5401119001
Trypsin-EDTA 0.25%	ThermoFisher	Cat# 25200056
Rat Collagen I	RnDSystems	Cat# 3447-020-01
Mammary Epithelial Cell Growth Medium	Promocell	Cat# c-21010
Mammary Epithelial Cell Growth Supplement	Sciencell	Cat# 7652
Doxycycline Hyclate	Sigma	Cat# D9891
Advanced DMEM/F12	Gibco	N/A
Primocin	Invivogen	Cat# ant-pm-05
1% GlutaMAX	Gibco	Cat# 35050038
N-Acetylcysteine	Sigma	Cat# A9165
1% B27 supplement	Gibco	Cat# 17504044
Epidermal Growth Factor (EGF)	Peprotech	Cat# GMP100-15
Noggin	Peprotech	Cat# 120-10C
A83-01	Tocris Bioscience	Cat# 2939
Gentle Cell Dissociation Reagent	StemCellTechnologies	Cat# 100-0485
DMEM FluoroBrite™	ThermoFisher	Cat# A1896701
Sodium Pyruvate 100mM	ThermoFisher	Cat# 11360070
MEM NEAA 100X	ThermoFisher	Cat# 11140035
L-Glutamine 200 mM	ThermoFisher	Cat# 25030123
SirActin	Spyrochrome AG	Cat# SC001
Hoechst-33342	ThermoFisher	Cat# H1399
FM4-64	ThermoFisher	Cat# T13320
BODIPY 493/503	ThermoFisher	Cat# D3922
Cellbanker 2 cryo-preserving medium	AMSBIO	Cat# 11891
Ficoll 70.000 MW	Merck KgaA	Cat# F2878

**Deposited data**		

FIB-SEM volume imaging	This study	EMPIAR-11380
Confocal fluorescence microscopy	This study	S-BIAD610

**Experimental models: Cell lines**		

BT-474 HTB-20™	ATCC	https://www.atcc.org/products/htb-20
Patient-derived colorectal cancer cell line (HT6) was established from a biosample provided by the Lungbiobank Heidelberg [member of the biomaterial bank Heidelberg (BMBH)] and established as described in the method details	BMBH	N/A

**Experimental models: Organisms/strains**		

Primary mouse mammary epithelial cells isolated from experimental line TetO-MYC/TetO-Neu/MMTV-rtTA/ R26-H2B-mCherry in FVB background	Transgenic strains^75^^,^^76^^,^^77^	See method details for additional information and literature references

**Software and algorithms**		

Fiji	Schneider et al.^78^	https://fiji.sc/
Fiji pluging create montage	Fiji macro by Patrice Mascalchi	https://github.com/AiviaCommunity/ImageJ-Macros-Utilities/blob/master/CreateMontageWithTime_2.0.ijm
Imaris 9.6.0	Bitplane	https://imaris.oxinst.com/
Matplotlib 3.5.0	Hunter et al.^79^	https://matplotlib.org/
Python 2.7	N/A	https://www.python.org/
IMOD v4.9	Kremer et al.^80^	https://bio3d.colorado.edu/imod/
Dragonfly 2021.3	ORS	https://www.theobjects.com/index.html
Biorender	Graphical abstract created with Biorender	https://biorender.com/
SerialFIB	Klumpe et al.^81^	https://github.com/sklumpe/SerialFIB
ChimeraX	Pettersen et al.^82^	https://www.cgl.ucsf.edu/chimerax/
SerialEM v3.7.2	Schorb et al.^83^	https://bio3d.colorado.edu/SerialEM/
Adobe Illustrator	Adobe Inc.	https://www.adobe.com/products/illustrator.html
Leica Application Suite X	Leica Microsystems, GmbH	https://www.leica-microsystems.com/products/microscope-software/p/leica-las-x-ls/
Zen Black	Carl Zeiss AG	https://www.micro-shop.zeiss.com/de/de/softwarefinder/software-categories/zen-black/

**Other**		

Glass bottom dishes	Mattek	Cat# P35G-1.5-10-C
Copper gold coated HPF carriers with 200 μm recess	Wohlwend GmbH http://www.wohlwend-hpf.ch/	Cat# 662
Multiwell dish 24 wells	ThermoFisher	Cat# 142475
Coverslip 12mm	Th. Geyer GmbH & Co. KG.	Cat# 9161064
Cell culture dishes 35 and 10mm	Greiner Bio One International	Cat# 627860

### Resource availability

#### Lead contact

Further information and requests for resources and reagents should be directed to and will be fulfilled by the lead contact Julia Mahamid (julia.mahamid@embl.de).

#### Materials availability

This study did not generate new unique reagents.

### Experimental model and subject details

Experimental animals: Primary mammary gland 3D cell cultures generated in this work originate from 8–12 weeks old female mice. TetO-MYC/MMTV-rtTA^75^ and TetO-Neu/MMTV-rtTA^76^ mouse strains were bred to obtain TetO-MYC/TetO-Neu/MMTV-rtTA animals. A reporter R26-H2B-mCherry mouse strain line^77^ (RIKEN, CDB0239K) was crossed in to establish the experimental line TetO-MYC/TetO-Neu/MMTV-rtTA/R26-H2B-mCherry in FVB background and were used for experimental procedures. The animals were maintained in individually ventilated plastic cages (Tecniplast) in an air-conditioned (temperature 22 °C ± 2 °C, humidity 50% ± 10%) and light-controlled room (illuminated from 07:00 to 19:00 h). Mice were fed 1318 P autoclavable diet (Altromin, Germany) ad libitum. All animal care and procedures performed in this study conformed to the EMBL Guidelines for the Use of Animals in Experiments and were reviewed and approved by the Institutional Animal Care and Use Committee (IACUC) (approval # MJ160070). All efforts were made to use the minimal possible number of animals in accordance with Russell and Burch’s (1959) principle of (3Rs) reduction and highest ethical standards. The work was approved by the IACUC (Institutional Animal Care and Use Committee, approval #160070 to MJ).

Human subjects: HT6 cell line derived from a metastatic lesion from a single patient diagnosed with colorectal cancer was use to generate 3D cell cultures. A metastatic colorectal cancer lesion was surgically removed, dissected from lung tissue and experimentally used. The patient biosample was provided by Lungbiobank Heidelberg member of the biomaterial bank Heidelberg (BMBH) in accordance with the regulations of the BMBH and the approval of the ethics committee of the University of Heidelberg (study S-270/2001 – biobank vote). Patients give a broad consent for biomaterial and data to the Lungenbiobank Heidelberg (member of the NCT- biobank and the BMBH).

### Method details

#### 3D cultures

All sample types described hereafter share culture conditions and handling in both HPF carriers and standard TC dishes, only differing in the Matrigel volume and the carrier type.

#### Primary mouse mammary epithelial organoid culture

Mammary glands were dissected from female virgin mice and collected in a 15 ml falcon tube. For dissociation of the tissue, the mammary glands were digested overnight at 37 ºC and 5% CO_2_ in a loosely capped 50 ml falcon tube with 5 ml of DMEM/F12 supplemented with 25 mM HEPES, 1% Penicillin Streptomycin solution, 750 units of Collagenase Type III and 20 μg of Liberase TM. After 16 h digestion, cells were washed with DMEM/F12 supplemented with 25 mM HEPES, 1% Penicillin Streptomycin solution. Pellet was then trypsinized with 5 ml of 0.25% Trypsin-EDTA to obtain single cells following the published protocol.^84^ To generate 3D cultures, a master mix of Matrigel Growth Factors Reduced, Rat Collagen I, and PBS was prepared on ice following 4:1:1 proportion. The cell suspension and matrix mix were combined to obtain a concentration of 30,000 cells/100 μl of master mix. Volumes of 1 μl were seeded in the 200 μm deep HPF carriers, previously sterilised with 70% Ethanol and fixed with Matrigel to an 18 mm glass coverslip. Then, each carrier was placed into a well of a 24-well plate to allow the gels to polymerize at 37 ºC with 5% CO_2_. After polymerization, each well was supplied with 1 ml of Mammary Epithelial Cell Growth Medium supplemented with Mammary Epithelial Cell Growth supplement and incubated at 37 ºC in a humidified atmosphere with 5% CO_2_. For induction of oncogene expression in the organoids, Doxycycline Hyclate was diluted into the growth medium at 600 ng/ml after 4 days of 3D cell culture. Growth media were exchanged every other day.

#### Colorectal cancer organoid culture

Metastatic lesion from a patient diagnosed with metastatic colorectal cancer was surgically removed and dissected from lung tissue. A tumor fragment of a minimum of 100 mm^2^ was used for the establishment of primary organoid culture. Briefly, tissue was mechanically dissociated using scalpel followed by pipetting through a 10 ml pipette in a basal medium Advanced DMEM/F12, Primocin 50 μg/ml, 1% GlutaMAX, 1% HEPES, 1% Penicillin Streptomycin solution, N-Acetylcysteine 1.25 mM supplemented with 1% B27 supplement. Next, the cell suspension was enzymatically digested for 2 h at 37 ºC in basal medium supplemented with Liberase DH (final concentration 0.28 Wünsch units/ml). The cell suspension was then filtered through 100 μm and 40 μm cell strainer. Single cells were seeded in Growth-Factor Reduced Matrigel mixed with PBS (4:1) adjusted to a concentration of 17,000 cells/μl and cultured in culture medium Advanced DMEM/F12, Primocin 50 μg/ml, 1% GlutaMAX, 1% HEPES, penicillin 100 U/ml and streptomycin 100 μg/ml, N-Acetylcysteine 1.25 mM supplemented with 1% B27 supplement, 50 ng/ml Epidermal Growth Factor (EGF), 100 ng/ml Noggin and 500 nM A83-01. Organoids were passaged with Gentle Cell Dissociation Reagent according to manufacturer instructions. For imaging experiments, organoids from passages 4-10 were seeded in HPF carriers using 2 μl of organoid suspension in Matrigel/PBS (6:1).

#### BT-474 spheroids

BT-474 human cell line was obtained from American Type Culture Collection. Cells were grown in DMEM 1x 4.5 g/L D-glucose FluoroBrite medium supplemented with 10% inactivated FBS, 1% HEPES 1 M, 1% Sodium Pyruvate 100 mM, 1% MEM NEAA 100X, 1% L-Glutamine 200 mM, 1% Penicillin Streptomycin solution and passaged using 0,05% Trypsin-EDTA. For spheroid formation and imaging experiments, cells were seeded in HPF carriers in Growth-Factor Reduced Matrigel mixed with PBS (6:1) at 500 cells/μl.

#### Immunofluorescent staining

Organoid cell culture was performed in HPF carriers which were then transferred to a deactivated clear glass vial, fixed with 4% PFA for 5 min followed by three washes of PBS. To prevent nonspecific antibody binding, the HPF carriers were incubated with 10% goat serum for 2 h at room temperature. Primary antibody incubation was performed overnight at 4 ºC. Afterward, HPF carriers were washed with PBS three times for 10 min, and incubated with secondary antibodies and DAPI (1:1000). Samples were mounted using Prolong Gold with DAPI. The following primary antibodies were used in this study: Rabbit anti c-MYC (1:800), Rabbit Anti ZO-1 (1:400), Mouse Anti E-cadherin (1:200), Rat Anti Alpha6-integrin (1:100), Mouse Anti Cytokeratin 8 (1:100), Mouse anti Cytokeratin 14 (1:200) Rabbit anti Cytokeratin 20 (1:200), detected by secondary antibodies Alexa 488 anti rabbit (1:800), Alexa 568 anti mouse (1:800), Alexa 647 anti rat (1:800). Mounts were imaged in HPF carriers on a Leica SP5 confocal microscope using a 63x 1.2 NA water immersion lens and the LAS AF imaging software. HPF carriers were mounted facing the objective on top of a Nunc Lab-Tek II chambered 1.5 borosilicate coverglass.

#### Stereomicroscopy and widefield transmission imaging

The 3D cell cultures in HPF carriers were maintained in 24-well plates and imaged in a Leica M125 C Stereomicroscope in dark field mode to enhance the HPF carriers contrast against the background. 3D cell culture grown in gels in TC-dishes over the time-course of the experiment were imaged using the widefield high-throughput Olympus ScanR microscope in transmission mode. Each well of the 24-well plate was imaged using 9 ROIs per well, with 21 Z-stacks (100 μm of scanning step in Z). Images were acquired with 4x UplanSApo 0.16 NA Air objective in an environmental chamber at standard cell culture conditions (37 °C, 5% CO_2_). Projections of z-stacks and image stitching were done using Fiji.^78^

#### Live-cell confocal imaging

Here, we refer to single time-points acquisitions and not to time-series commonly known as 4D imaging. For live cell imaging, cultures in HPF carriers were washed with PBS once, transferred to a 35 mm or 10 mm diameter cell culture dish. For samples devoid of genetic fluorescent tags, the HPF carriers were incubated for 20 min with the desired live dyes diluted in the growth medium to the following final concentrations: SiR-actin 100 μM, Hoechst-33342 10 μM, FM4-64 2 μM and BODIPY 493/503 1 μM. The culture dish was connected to the microscope objective by a drop of deionized water, and HPF carriers were flipped with the recess facing the microscope objective lens. Live cell imaging was performed at 37°C and 5% CO_2_ with a Zeiss LSM 780 NLO (Carl Zeiss AG, Jena, Germany) equipped with a LD LCI Plan-Apochromat 25x 0.8 NA water immersion objective. To image the whole HPF carrier volume and locate single organoids, we first detected cell nuclei marked with Hoechst-33342 and acquired z-stacks with 6x6 tiles of 512^2^ pixels with 10% overlap, and 10 μm z-step. The final montage was stitched using ZEN-black software. For single organoids, we acquired z-stacks of 1024^2^ pixels at different z-steps ranging from 1.0 to 1.8 μm.

#### High-pressure freezing, freeze-substitution, and two-photon laser branding

We followed the procedure described in Ronchi et al.^30^ Briefly, high-pressure freezing was performed in HPF carriers with an HPM 010 (AbraFluid AG, Rebstein, Switzerland). When necessary, before high-pressure freezing, the carriers containing the 3D cell cultures were dipped for 1 min either directly in Cellbanker 2 cryo-preserving medium or in 20% Ficoll 70.000 MW diluted in Mammary Epithelial Cell Growth Medium. Next, freeze-substitution was performed with 0.1% uranyl acetate (UAc) in acetone. After 72 h incubation at -90 °C, the temperature was increased to allow the reaction of UAc with the biological material. The samples were then rinsed with pure acetone before infiltration of the resin lowicryl HM20. The resin was polymerized with UV at -25 °C. The resin-embedded samples were transferred into 35 or 10 mm cell culture dish with water as immersion medium and imaged at an inverted Zeiss LSM 780 NLO microscope equipped with a 25x Plan-Apochromat 25x 0.8 NA Imm Korr DIC multi immersion objective lens. Surface branding was performed with the 2-photon Coherent Chameleon Ultra II Laser (Coherent Inc, Santa Clara, USA) of the Zeiss LSM 780 NLO microscope and the “bleaching” function of ZEN black software.

#### Cryo-confocal microscopy

Confocal stack acquisition under cryo-conditions was carried out with Leica TCS SP8 upright microscope (Leica microsystems CMS GmbH, Mannheim, Germany), controlled by Leica Application Suite X 3.5.5.19976 software. The microscope was equipped with a cryo-stage, insulated HC PL APO 50x 0.90 NA DRY and HC PL FLUOTAR 50x 0.80 NA objectives, and Leica DFC365 FX camera. We found the Leica HC PL FLUOTAR 50x 0.80 Numerical Aperture (NA) objective to provide the best compromise for high NA and long working distance (1.02 mm) to account for HPF carriers’ depth (> 100 μm) and large size of the organoids (> 100 μm). The high-pressure frozen organoids in HPF carriers were inserted into the shuttle (Leica microsystems CMS GmbH, Mannheim, Germany) with the sample side facing the objective. All sample loading and transfer operations were conducted in the liquid nitrogen vapor phase using a dedicated loading/transfer unit (Leica microsystems CMS GmbH, Mannheim, Germany). The loading and transfer steps were carried out in a humidity-controlled room (10%) to minimize ice contamination on the sample. After the sample was deposited on the microscope stage pre-cooled to -195° C, the approximate z-position of the sample surface was determined in wide-field illumination mode. Using Matrix MAPS+CLEM2 application within the Matrix Screener module, 100 μm deep (steps of 2 μm) z-stack tiles (x/y with 20% overlapping) were acquired to produce a stitched 3D overview image of the whole carrier at the desired wavelengths (Leica Application Suite X 3.5.5.19976). This helped to determine ROIs for subsequent confocal stacks acquisition. Confocal stacks were acquired in two channels: 405 nm at 50% power Diode laser (HyD1 detector 410 nm – 504 nm) and 552 nm at 50% power OP SL laser (HyD 3 detector 660 nm – 778 nm). Pinhole 1 AU, zoom 1, pixel format 1056x1056, pixel size in x,y = 0.22 μm, in z = 0.372 μm, bidirectional scan at 200 Hz. Z-stack of 50 μm depth were acquired with a z-step system optimized for the 405 nm wavelength. Confocal stacks were processed with the Lightning deconvolution software module (Leica Application Suite X 3.5.5.19976) at default settings and a number of iterations set to 5 for both channels. After the acquisition, the HPF carriers were retrieved under cryo-conditions for further processing.

#### FIB-SEM

For resin-embedded samples after confocal imaging and branding for targeting, the blocks were mounted on a SEM stub using silver conductive epoxy resin. The samples were gold sputtered with a Quorum Q150R S coater. FIB-SEM imaging was performed on a Zeiss CrossBeam XB540 or XB550 (Carl Zeiss AG Jena, Germany). Briefly, platinum was deposited over the area marked by the laser branding. Auto-tuning marks were milled on the platinum surface and highlighted with carbon. Large trenches were milled with 30 nA FIB current and surface polished with 7 or 15 nA. Precise milling during the run was achieved with currents of either 700 pA or 1.5 nA. For all experiments, the SEM imaging was done with an acceleration voltage of 1.5 kV and a current of 700 pA, using a back-scattered electron detector. For single cells within organoids, data were acquired at 10 nm isotropic (x,y,z) sampling. For the acquisition of entire organoids, voxels of 15x15x20 nm^3^ were found to be the best compromise between the achievable resolution/field-of-view ratio and milling stability.

#### Thin sectioning TEM

Blocks prepared as described above were sectioned with an ultramicrotome Leica UC7 (Leica microsystems CMS GmbH, Mannheim, Germany) and 70 nm sections were collected on formvar coated slot grids. TEM images were acquired without post-staining using a Jeol 2100 Plus operated at 120 kV (Jeol Ltd, Tokyo, Japan). The organoids were identified at 400x mag (32 nm pixel size). Subsequently, single organoids were imaged by fitting a montage of tiles at 2000x mag (6.7 nm pixel size) using SerialEM.^83^ The montages were stitched with the command justblend and, where necessary, manually corrected with Midas, both implemented in Imod.^80^ To estimate the ultrastructural preservation of organoids, six to ten organoids per condition were imaged by tiling multiple image spots: BT-474 spheroids (1619 tiles), patient-derived colorectal cancer organoids (823 tiles), doxycycline-induced tumorigenic (430 tiles) and healthy (596 tiles) mouse mammary gland organoids, and healthy mouse mammary gland organoids high-pressure frozen in presence of Cellbanker 2 cryo-preserving medium or Ficoll (70.000 MW) 20% (450 tiles). Ultrastructural preservation was scored by counting tiles with evident freeze damage versus tiles devoid of it (see also Figure S3A). All the statistical analysis and plots were performed with Python and Matplotlib^79^ respectively.

#### Deep learning automatic volume segmentation and data mining

ORS Dragonfly 2021.3 and subsequent versions were used to train neural networks for automatic segmentation of FIB-SEM data. Dragonfly was installed on a virtual machine workstation with Windows 10 pro mounting an IntelI XI(R) Gold 6226R CPU 2.90 GHz with 128 GB RAM and an Nvidia V100S card with 30 GB RAM. For pre-processing, the raw FIB-SEM frames were aligned with the Scale-Invariant Feature Transform (SIFT) algorithm^85^ implemented as a macro in Fiji (https://imagej.net/plugins/linear-stack-alignment-with-sift),^78^ allowing only image transformation by translation. Next, the images were cropped to exclude pixels without signal outside imaged areas. FIB-SEM datasets containing few cells acquired with 10 nm isotropic resolution were processed as such, while whole organoid acquisitions (15x15x20 nm resolution) were binned twice for computational efficiency. CNN models were prepared with the 2.5D-UNet architecture and the following design: depth level 6 with 7 slices for CNNs for microvillar actin bundles, nuclei, cell bodies and cell boundaries; depth level 6 with 5 slices for cell junctions and depth level 5 with 5 slices for mitochondria. Next, using the segmentation wizard module, ground truth data was manually annotated from 2 to 5 subframes of each dataset and used to train each CNN with 10-fold data augmentation by: flipping vertically and horizontally, rotation up to 180°, shear up to 2° and scaling between 90 and 110%. For training, patches ranging between 128^2^ and 512^2^ pixels were used depending on the size of the objects. These initially trained CNNs models were then used to segment 5 to 10 whole frames from each dataset, where errors were corrected manually. The learning convergence was followed by monitoring the dice-score plateauing within 15-30 epocs and scrutinising at each epoc the updated inference on a reference frame not used for training. Then, the model weights were reset and the CNNs were re-trained until learning convergence (typically < 50 epocs) using the whole selected frames without data augmentation this time. Finally, the CNNs were run over all the datasets to segment cell bodies and subcellular structures. Because each acquisition includes different features and noise levels, for consistent results, it was necessary to re-train the CNNs on each acquisition. However, the procedure was much faster because i) pre-trained CNNs models learn much quicker, *i.e.* just half the epocs were required; ii) the pre-trained CNN was used to directly segment only a couple of input training frames which required little manual correction. Subsequently, the re-trained CNNs were used to segment the new datasets.

To compute the nearest-neighbour analysis, the instance segmentation of the organelle/structure of interest was first converted to “multi-ROI”. Here, the global instance segmentation is split into single 3D objects based on their 3D connected components: objects not connected to each other are considered as an independent item belonging to a global instance segmentation. Due to the lower resolution in z of the FIB-SEM images used for segmentation (40 nm), objects closer than 50 nm were excluded by the count. Then, nearest-neighbour was calculated using the “Find minimum distance between objects” option. To compute local density maps of segmentations, the “Bone analysis” wizard was used to estimate volume fractions. After optimizing parameters for resolving power and computing time, each FIB-SEM acquisition was sampled using a sphere of 1 μm diameter (∼ 4 μm^3^ sampling volume) and scanning the volume using 0.25 μm step size. The resulting density maps were colored and displayed in Dragonfly. Plots were computed with Matplotlib.^79^

#### Data processing and rendering

Unless otherwise specified, all microscopy data were processed with Fiji.^78^ Where necessary, the contrast of electron microscopy data was enhanced using the Fiji plugin Enhance Local Contrast (CLAHE) using the following parameters: blocksize 63, 256 histogram bins and maximum slope of 2.0. When necessary, before contrast enhancement, FIB-SEM volumes were corrected for curtaining effects by applying wavelet decomposition^86^ implemented in the open-source software SerialFIB.^81^ Here, coif3 type vertical wavelets with a decomposition sigma level of 8 and a sigma gaussian for vertical stripes dampening of 6 were used. All FIB-SEM and segmentation rendering and videos were generated with ChimeraX.^82^ For light microscopy data 3D rendering, Imaris 9.6.0 with either “blend” or “shadow projection” rendering modes was used. Videos with multiple channels synchronized were made with the Fiji macro by Patrice Malscalchi (https://github.com/AiviaCommunity/ImageJ-Macros-Utilities/blob/master/CreateMontageWithTime_2.0.ijm). Schematics were created with Biorender (Biorender.com) and Adobe illustrator.

### Quantification and statistical analysis

Quantification of the ultrastructural preservation of organoids is displayed as box plots in Figure 4F (Related to Figures S3–S5). Orange line indicates the median; box, 25-75% interval; wiskers, minimum and maximum. Quantification was based on scoring individual tiles following the visual inspections of TEM tiled acquisitions of negatively stained thin sections (see method details): human breast spheroids (BT474, n=7 spheroids, 1619 examined tiles), human colorectal cancer organoids (Colon, n=11 organoids, 823 examined tiles), doxycycline-induced tumorigenic (Mouse Ind, n=8 organoids, 430 examined tiles), healthy (mouse NI, n=8 organoids, 596 examined tiles) mouse mammary gland organoids that were additionally supplemented with cryo-protectants (Cryo-protectants, n=6 organoids, 450 examined tiles). In Figure 4G, a possible relationship between organoid cell packing and ultrastructural preservation was investigated. The cellular density (as the ratio between organoid area and number of cells, see method details) was plotted versus the ultrastructural preservation per organoid (as a percentage of tiles showing ultrastructural preservation from the TEM thin sections quantified in Figure 4F). Each data point represents one organoid. A Pearson correlation coefficient of R=0.39 was calculated and indicates weak correlation between cell packing and ultrastructural preservation. Methods to determine whether the data met assumptions of the statistical approach were not employed.

The statistical analysis of FIB-SEM segmentations (Figure 7) involved calculation of nuclear sphericity and cell-junctions nearest neighbour. Nuclear sphericity was measured in Dragonfly as:

$$\frac{{\left(6\pi^{\frac{1}{2}}V_{P}\right)}^{2/3}}{A_{P}}$$

Where V_p_ is the volume of the particle and A_p_ is the surface area of the particle. In this case, the surface area is computed using the Lindblad surface area estimator method.

For nearest neighbour analysis, we computed the nearest linear distances between the coordinates of individual cell junctions segmentations using their centroid, instead of volume boundaries to reduce the bias due to possible incorrect segmentation.

Boxes within the violins represent 25-75% data interval, and diamonds indicate medians. Number of cells (and nuclei), cellular volume fractions of nuclei, and cell junction numbers for the different organoid areas are listed in Figure 7G.

Additional details of the quantification and all statistical analyses are included in figure captions or the relevant sections of method details.

## Data Availability

FIB-SEM data and corresponding fluorescence light microscopy of human BT-474 spheroids and patient-derived colorectal cancer organoids are available on EMPIAR under accession code EMPIAR-11380 (https://doi.org/10.6019/EMPIAR-11380) and Bioimage Archive under accession code S-BIAD610 (https://www.ebi.ac.uk/biostudies/bioimages/studies/S-BIAD610). Any additional information required to re-analyze the data reported in this paper is available from the lead contact upon request.

## Supplemental information

Document S1. Figures S1–S6

Document S2. Article plus supplemental information

## References

[1] Baker B.M., Chen C.S. (2012). Deconstructing the third dimension – how 3D culture microenvironments alter cellular cues. J. Cell Sci..

[2] Kretzschmar K., Clevers H. (2016). Organoids: modeling development and the stem cell niche in a dish. Dev. Cell.

[3] Knouse K.A., Lopez K.E., Bachofner M., Amon A. (2018). Chromosome segregation fidelity in epithelia requires tissue architecture. Cell.

[4] Kapałczyńska M., Kolenda T., Przybyła W., Zajączkowska M., Teresiak A., Filas V., Ibbs M., Bliźniak R., Łuczewski Ł., Lamperska K. (2018). 2D and 3D cell cultures – a comparison of different types of cancer cell cultures. Arch. Med. Sci..

[5] Zanoni M., Cortesi M., Zamagni A., Arienti C., Pignatta S., Tesei A. (2020). Modeling neoplastic disease with spheroids and organoids. J. Hematol. Oncol..

[6] Lancaster M.A., Huch M. (2019). Disease modelling in human organoids. Dis. Model. Mech..

[7] Hughes C.S., Postovit L.M., Lajoie G.A. (2010). Matrigel: A complex protein mixture required for optimal growth of cell culture. Proteomics.

[8] Koledova Z. (2017). 3D cell culture: an introduction. Methods Mol. Biol..

[9] Shamir E.R., Ewald A.J. (2014). Three-dimensional organotypic culture: experimental models of mammalian biology and disease. Nat. Rev. Mol. Cell Biol..

[10] Fatehullah A., Tan S.H., Barker N. (2016). Organoids as an in vitro model of human development and disease. Nat. Cell Biol..

[11] Simian M., Bissell M.J. (2017). Organoids: A historical perspective of thinking in three dimensions. J. Cell Biol..

[12] Hof L., Moreth T., Koch M., Liebisch T., Kurtz M., Tarnick J., Lissek S.M., Verstegen M.M.A., van der Laan L.J.W., Huch M. (2021). Long-term live imaging and multiscale analysis identify heterogeneity and core principles of epithelial organoid morphogenesis. BMC Biol..

[13] Day R.N., Davidson M.W. (2009). The fluorescent protein palette: tools for cellular imaging. Chem. Soc. Rev..

[14] Keshara R., Kim Y.H., Grapin-Botton A. (2022). Organoid imaging: seeing development and function. Annu. Rev. Cell Dev. Biol..

[15] Tomer R., Ye L., Hsueh B., Deisseroth K. (2014). Advanced CLARITY for rapid and high-resolution imaging of intact tissues. Nat. Protoc..

[16] Dekkers J.F., Alieva M., Wellens L.M., Ariese H.C.R., Jamieson P.R., Vonk A.M., Amatngalim G.D., Hu H., Oost K.C., Snippert H.J.G. (2019). High-resolution 3D imaging of fixed and cleared organoids. Nat. Protoc..

[17] Ertürk A., Becker K., Jährling N., Mauch C.P., Hojer C.D., Egen J.G., Hellal F., Bradke F., Sheng M., Dodt H.-U. (2012). Three-dimensional imaging of solvent-cleared organs using 3DISCO. Nat. Protoc..

[18] Knott G., Genoud C. (2013). Is EM dead?. J. Cell Sci..

[19] Peddie C.J., Genoud C., Kreshuk A., Meechan K., Micheva K.D., Narayan K., Pape C., Parton R.G., Schieber N.L., Schwab Y. (2022). Volume electron microscopy. Nat. Rev. Methods Primers.

[20] Heymann J.A.W., Hayles M., Gestmann I., Giannuzzi L.A., Lich B., Subramaniam S. (2006). Site-specific 3D imaging of cells and tissues with a dual beam microscope. J. Struct. Biol..

[21] Giannuzzi L.A., Stevie F.A., Giannuzzi B.L.A., Stevie F.A. (2005).

[22] Narayan K., Subramaniam S. (2015). Focused ion beams in biology. Nat. Methods.

[23] Kukulski W., Schorb M., Welsch S., Picco A., Kaksonen M., Briggs J.A.G. (2011). Correlated fluorescence and 3D electron microscopy with high sensitivity and spatial precision. J. Cell Biol..

[24] Ganeva I., Kukulski W. (2020). Membrane architecture in the spotlight of correlative microscopy. Trends Cell Biol..

[25] Mollenhauer H.H. (1991). Artifacts caused by dehydration and epoxy embedding in TEM. Microsc. Res. Tech..

[26] Dubochet J., McDowall A.W. (1981). Vitrification of pure water for electron microscopy. J. Microsc..

[27] Moor H. (1987). Cryotechniques in Biological Electron Microscopy.

[28] Kellenberger E. (1987). Cryotechniques in Biological Electron Microscopy.

[29] Nixon S.J., Webb R.I., Floetenmeyer M., Schieber N., Lo H.P., Parton R.G. (2009). A single method for cryofixation and correlative light, electron microscopy and tomography of zebrafish embryos. Traffic.

[30] Ronchi P., Machado P., D’imprima E., Mizzon G., Best B.T., Cassella L., Schnorrenberg S., Montero M.G., Jechlinger M., Ephrussi A. (2021).

[31] Jechlinger M. (2015). Organotypic culture of untransformed and tumorigenic primary mammary epithelial cells. Cold Spring Harb. Protoc..

[32] Triffo W.J., Palsdottir H., McDonald K.L., Lee J.K., Inman J.L., Bissell M.J., Raphael R.M., Auer M. (2008). Controlled microaspiration for high-pressure freezing: a new method for ultrastructural preservation of fragile and sparse tissues for TEM and electron tomography. J. Microsc..

[33] Alladin A., Chaible L., Garcia del Valle L., Sabine R., Loeschinger M., Wachsmuth M., Hériché J.-K., Tischer C., Jechlinger M. (2020). Tracking cells in epithelial acini by light sheet microscopy reveals proximity effects in breast cancer initiation. eLife.

[34] Kummar S., Fogarasi M., Canova A., Mota A., Ciesielski T. (2002). Cytokeratin 7 and 20 staining for the diagnosis of lung and colorectal adenocarcinoma. Br. J. Cancer.

[35] Florian S., Iwamoto Y., Coughlin M., Weissleder R., Mitchison T.J. (2019). A human organoid system that self-organizes to recapitulate growth and differentiation of a benign mammary tumor. Proc. Natl. Acad. Sci. USA.

[36] Shimokawa M., Ohta Y., Nishikori S., Matano M., Takano A., Fujii M., Date S., Sugimoto S., Kanai T., Sato T. (2017). Visualization and targeting of LGR5 + human colon cancer stem cells. Nature.

[37] Biel S.S., Kawaschinski K., Wittern K.-P., Hintze U., Wepf R. (2003). From tissue to cellular ultrastructure: closing the gap between micro- and nanostructural imaging. J. Microsc..

[38] Pfeiffer S., Vielhaber G., Vietzke J.-P., Wittern K.-P., Hintze U., Wepf R. (2000). High-pressure freezing provides new information on human epidermis: simultaneous protein antigen and lamellar lipid structure preservation. Study on human epidermis by cryoimmobilization. J. Invest. Dermatol..

[39] Sitte H., Edelmann L., Neumann K. (1987). Cryotechniques in Biological Electron Microscopy.

[40] Möbius W., Cooper B., Kaufmann W.A., Imig C., Ruhwedel T., Snaidero N., Saab A.S., Varoqueaux F. (2010). Electron microscopy of the mouse central nervous system. Methods Cell Biol..

[41] Smeets M., Bieber A., Capitanio C., Schioetz O., van der Heijden T., Effting A., Piel É., Lazem B., Erdmann P., Plitzko J. (2021). Integrated cryo-correlative microscopy for targeted structural investigation in situ. Micros. Today.

[42] Almendro V., Marusyk A., Polyak K. (2013). Cellular heterogeneity and molecular evolution in cancer. Annu. Rev. Pathol..

[43] Fais S., Overholtzer M. (2018). Cell-in-cell phenomena in cancer. Nat. Rev. Cancer.

[44] Overholtzer M., Mailleux A.A., Mouneimne G., Normand G., Schnitt S.J., King R.W., Cibas E.S., Brugge J.S. (2007). A nonapoptotic cell death process, entosis, that occurs by cell-in-cell invasion. Cell.

[45] te Boekhorst V., Friedl P. (2016). Plasticity of cancer cell invasion—mechanisms and implications for therapy. Adv. Cancer Res..

[46] Huang Z., Zhang Z., Zhou C., Liu L., Huang C. (2022). Epithelial–mesenchymal transition: the history, regulatory mechanism, and cancer therapeutic opportunities. MedComm. MedComm (2020).

[47] Fawcett D.W. (1966). An atlas of fine structure. The cell. Its organelles and inclusions. Ann. Intern. Med..

[48] Schmidt R., Wurm C.A., Punge A., Egner A., Jakobs S., Hell S.W. (2009). Mitochondrial cristae revealed with focused light. Nano Lett..

[49] Egner A., Jakobs S., Hell S.W. (2002). Fast 100-nm resolution three-dimensional microscope reveals structural plasticity of mitochondria in live yeast. Proc. Natl. Acad. Sci. USA.

[50] Skinner B.M., Johnson E.E.P. (2017). Nuclear morphologies: their diversity and functional relevance. Chromosoma.

[51] Zink D., Fischer A.H., Nickerson J.A. (2004). Nuclear structure in cancer cells. Nat. Rev. Cancer.

[52] Otani T., Nguyen T.P., Tokuda S., Sugihara K., Sugawara T., Furuse K., Miura T., Ebnet K., Furuse M. (2019). Claudins and JAM-A coordinately regulate tight junction formation and epithelial polarity. J. Cell Biol..

[53] Beutel O., Maraspini R., Pombo-García K., Martin-Lemaitre C., Honigmann A. (2019). Phase separation of zonula occludens proteins drives formation of tight junctions. Cell.

[54] Odenwald M.A., Choi W., Kuo W.T., Singh G., Sailer A., Wang Y., Shen L., Fanning A.S., Turner J.R. (2018). The scaffolding protein ZO-1 coordinates actomyosin and epithelial apical specializations in vitro and in vivo. J. Biol. Chem..

[55] Hötte K., Koch M., Hof L., Tuppi M., Moreth T., Verstegen M.M.A., van der Laan L.J.W., Stelzer E.H.K., Pampaloni F. (2019). Ultra-thin fluorocarbon foils optimise multiscale imaging of three-dimensional native and optically cleared specimens. Sci. Rep..

[56] Huang Q., Garrett A., Bose S., Blocker S., Rios A.C., Clevers H., Shen X. (2021). The frontier of live tissue imaging across space and time. Cell Stem Cell.

[57] St Croix B.St., Kerbel R.S. (1997). Cell adhesion and drug resistance in cancer. Curr. Opin. Oncol..

[58] Dongre A., Weinberg R.A. (2019). New insights into the mechanisms of epithelial–mesenchymal transition and implications for cancer. Nat. Rev. Mol. Cell Biol..

[59] van Staalduinen J., Baker D., ten Dijke P., van Dam H. (2018). Epithelial–mesenchymal-transition-inducing transcription factors: new targets for tackling chemoresistance in cancer?. Oncogene.

[60] Vucetic M., Daher B., Cassim S., Meira W., Pouyssegur J. (2020). Together we stand, apart we fall: how cell-to-cell contact/interplay provides resistance to ferroptosis. Cell Death Dis..

[61] Pleguezuelos-Manzano C., Puschhof J., Rosendahl Huber A., van Hoeck A., Wood H.M., Nomburg J., Gurjao C., Manders F., Dalmasso G., Stege P.B. (2020). Mutational signature in colorectal cancer caused by genotoxic pks+ E. coli. Nature.

[62] Schmidt T.S.B., Raes J., Bork P. (2018). The human gut microbiome: from association to modulation. Cell.

[63] Yu L.C.-H. (2018). Microbiota dysbiosis and barrier dysfunction in inflammatory bowel disease and colorectal cancers: exploring a common ground hypothesis. J. Biomed. Sci..

[64] Kapteijn R., Shitut S., Aschmann D., Zhang L., Beer M. de, Daviran D., Roverts R., Akiva A., Wezel G.P. van, Kros A. (2022).

[65] Tischler J., Swank Z., Hsiung H.-A., Vianello S., Lutolf M.P., Maerkl S.J. (2022). An automated do-it-yourself system for dynamic stem cell and organoid culture in standard multi-well plates. Cell Rep. Methods.

[66] Decembrini S., Hoehnel S., Brandenberg N., Arsenijevic Y., Lutolf M.P. (2020). Hydrogel-based milliwell arrays for standardized and scalable retinal organoid cultures. Sci. Rep..

[67] Ntziachristos V. (2010). Going deeper than microscopy: the optical imaging frontier in biology. Nat. Methods.

[68] Helmchen F., Denk W. (2005). Deep tissue two-photon microscopy. Nat. Methods.

[69] Richter V., Rank M., Heinrich A., Schneckenburger H. (2022). Novel approaches in 3D live cell microscopy. Quantum Electron..

[70] Fei K., Zhang J., Yuan J., Xiao P. (2022). Present application and perspectives of organoid imaging technology. Bioengineering (Basel).

[71] Xu C.S., Hayworth K.J., Lu Z., Grob P., Hassan A.M., García-Cerdán J.G., Niyogi K.K., Nogales E., Weinberg R.J., Hess H.F. (2017). Enhanced FIB-SEM systems for large-volume 3D imaging. eLife.

[72] Heinrich L., Bennett D., Ackerman D., Park W., Bogovic J., Eckstein N., Petruncio A., Clements J., Pang S., Xu C.S. (2021). Whole-cell organelle segmentation in volume electron microscopy. Nature.

[73] Iudin A., Korir P.K., Somasundharam S., Weyand S., Cattavitello C., Fonseca N., Salih O., Kleywegt G.J., Patwardhan A. (2023). EMPIAR: the electron microscopy public image archive. Nucleic Acids Res..

[74] Ouyang W., Beuttenmueller F., Gómez-De-Mariscal E., Pape C., Burke T., Garcia-López-De-Haro C., Russell C., Moya-Sans L., De-La-Torre-Gutiérrez C., Schmidt D. (2022).

[75] D’Cruz C.M., Gunther E.J., Boxer R.B., Hartman J.L., Sintasath L., Moody S.E., Cox J.D., Ha S.I., Belka G.K., Golant A. (2001). c-MYC induces mammary tumorigenesis by means of a preferred pathway involving spontaneous Kras2 mutations. Nat. Med..

[76] Moody S.E., Sarkisian C.J., Hahn K.T., Gunther E.J., Pickup S., Dugan K.D., Innocent N., Cardiff R.D., Schnall M.D., Chodosh L.A. (2002). Conditional activation of Neu in the mammary epithelium of transgenic mice results in reversible pulmonary metastasis. Cancer Cell.

[77] Abe T., Kiyonari H., Shioi G., Inoue K.-I., Nakao K., Aizawa S., Fujimori T. (2011). Establishment of conditional reporter mouse lines at ROSA26 locus for live cell imaging. Genesis.

[78] Schneider C.A., Rasband W.S., Eliceiri K.W. (2012). NIH Image to ImageJ: 25 years of image analysis. Nat. Methods.

[79] Hunter J.D. (2007). Matplotlib: A 2D graphics environment. Comput. Sci. Eng..

[80] Kremer J.R., Mastronarde D.N., McIntosh J.R. (1996). Computer visualization of three-dimensional image data using IMOD. J. Struct. Biol..

[81] Klumpe S., Fung H.K.H., Goetz S.K., Zagoriy I., Hampoelz B., Zhang X., Erdmann P.S., Baumbach J., Müller C.W., Beck M. (2021).

[82] Pettersen E.F., Goddard T.D., Huang C.C., Meng E.C., Couch G.S., Croll T.I., Morris J.H., Ferrin T.E. (2021). UCSF ChimeraX: structure visualization for researchers, educators, and developers. Protein Sci..

[83] Schorb M., Haberbosch I., Hagen W.J.H., Schwab Y., Mastronarde D.N. (2019). Software tools for automated transmission electron microscopy. Nat. Methods.

[84] Jechlinger M., Podsypanina K., Varmus H. (2009). Regulation of transgenes in three-dimensional cultures of primary mouse mammary cells demonstrates oncogene dependence and identifies cells that survive deinduction. Genes Dev..

[85] Lowe D.G. (1999).

[86] Münch B., Trtik P., Marone F., Stampanoni M. (2009). Stripe and ring artifact removal with combined wavelet—Fourier filtering. Opt. Express.

